# Elevating EGFR-MAPK program by a nonconventional Cdc42 enhances intestinal epithelial survival and regeneration

**DOI:** 10.1172/jci.insight.135923

**Published:** 2020-08-20

**Authors:** Xiao Zhang, Sheila Bandyopadhyay, Leandro Pires Araujo, Kevin Tong, Juan Flores, Daniel Laubitz, Yanlin Zhao, George Yap, Jingren Wang, Qingze Zou, Ronaldo Ferraris, Lanjing Zhang, Wenwei Hu, Edward M. Bonder, Pawel R. Kiela, Robert Coffey, Michael P. Verzi, Ivaylo I. Ivanov, Nan Gao

**Affiliations:** 1 Department of Biological Sciences, Division of Life Sciences, School of Arts and Sciences, Rutgers University, Newark, New Jersey, USA.; 2 Department of Microbiology and Immunology, Vagelos College of Physicians and Surgeons, Columbia University, New York, New York, USA.; 3 Department of Genetics, Division of Life Sciences, School of Arts and Sciences, Rutgers University, New Brunswick, New Jersey, USA.; 4 Department of Pediatrics, University of Arizona, Tucson, Arizona, USA.; 5 Center for Immunity and Inflammation, Rutgers New Jersey Medical School, Newark, New Jersey, USA.; 6 Department of Mechanical and Aerospace Engineering, School of Engineering, Rutgers University, Piscataway, New Jersey, USA.; 7 Department of Pharmacology, Physiology and Neuroscience, Rutgers New Jersey Medical School, Newark, New Jersey, USA.; 8 Department of Pathology, University Medical Center of Princeton, Plainsboro, New Jersey, USA.; 9 Rutgers Cancer Institute of New Jersey, New Brunswick, New Jersey, USA.; 10 Division of Gastroenterology, Hepatology, and Nutrition, Department of Medicine, and Epithelial Biology Center, Vanderbilt University Medical Center, Nashville, Tennessee, USA.

**Keywords:** Gastroenterology, Stem cells, Adult stem cells, Mouse models

## Abstract

The regulatory mechanisms enabling the intestinal epithelium to maintain a high degree of regenerative capacity during mucosal injury remain unclear. Ex vivo survival and clonogenicity of intestinal stem cells (ISCs) strictly required growth response mediated by cell division control 42 (Cdc42) and Cdc42-deficient enteroids to undergo rapid apoptosis. Mechanistically, Cdc42 engaging with EGFR was required for EGF-stimulated, receptor-mediated endocytosis and sufficient to promote MAPK signaling. Proteomics and kinase analysis revealed that a physiologically, but nonconventionally, spliced Cdc42 variant 2 (V2) exhibited stronger MAPK-activating capability. Human CDC42-V2 is transcriptionally elevated in some colon tumor tissues. Accordingly, mice engineered to overexpress Cdc42-V2 in intestinal epithelium showed elevated MAPK signaling, enhanced regeneration, and reduced mucosal damage in response to irradiation. Overproducing Cdc42-V2 specifically in mouse ISCs enhanced intestinal regeneration following injury. Thus, the intrinsic Cdc42-MAPK program is required for intestinal epithelial regeneration, and elevating this signaling cascade is capable of initiating protection from genotoxic injury.

## Abstract 2

An intrinsic Cdc42-MAPK program is required for intestinal epithelial regeneration, and overexpressing Cdc42 variant 2 protects from genotoxic injury.

## Introduction

The intestinal epithelia are essential for nutrient absorption and survival of the host. Proper functioning of intestinal epithelial cells (IECs) relies on a signaling network that sustains continued cell production while simultaneously maintaining tissue integrity and retaining the crucial balance among progenitor and differentiated cell populations. Canonical Wnt and EGF signaling are 2 major growth pathways for intestinal stem cell (ISC) survival and renewal (1–4), while the BMP pathway drives formation of differentiated villus epithelia (5, 6). It is becoming increasingly evident that synergistic interplay of these pathways ensures a homeostasis balance, where crypt-based ISCs self-renew and produce various lineages of progenitors that, upon transit amplification, differentiate into mature IECs to offset programmed cell death (7, 8). Indispensability of Wnt, EGF, and BMP pathways for ISC function is unequivocally demonstrated in the enteroid culture system, in which an indefinite ISC renewal is sustained by Wnt and EGF pathway agonists along with a BMP pathway inhibitor, Noggin (9–11). Surprisingly, in contrast to enteroid models, intestinal epithelia in animals demonstrate remarkable plasticity displayed by stemness acquisition by quiescent and mature cell populations in response to injury-induced ISC loss (3, 12–21). The interactive complexity of intrinsic and extrinsic epithelial signaling that confers remarkable tissue plasticity remains both poorly defined and of important biomedical impact.

The cell division control 42 (Cdc42) is a Rho subfamily small GTPase with pleiotropic functions in cytoskeleton organization, cell polarity, and cell migration across eukaryotic species (22–24). Previously, we reported that elevated Cdc42 levels were observed in human and mouse intestinal tumors (25). Genome-wide association study identified a single nucleotide polymorphism in *CDC42* linked to irritable bowel syndrome (26). Genetic ablation of *Cdc42* in mouse IECs resulted in enteropathy resembling a lethal form of the pediatric disorder microvillus inclusion disease (27–30). The initial link between Cdc42 and ISC homeostasis was made based on the observation of abnormal organization of intestinal crypts in mice lacking epithelial Cdc42 (28). It remains unclear how Cdc42 controls ISC renewal and regeneration and whether modulating Cdc42 signaling networks may enhance ISC function.

Using proteomics and kinase assay screening, we identified the involvement of Cdc42 in endocytosis-associated EGF/EGFR/MAPK signaling. Elimination of Cdc42 in enteroids resulted in rapid apoptosis, coincident with lost EGF-stimulated EGFR vesicular traffic. We found that native Cdc42 splice variant 2 (V2) (31–36) has enhanced EGFR engagement and MAPK-activating capacity. Mice engineered to produce Cdc42-V2 in intestinal epithelia exhibited robust regenerative capability and mitigated injury-induced damage. These results provide fresh insight into the role of the epithelial intrinsic survival program in mediating protection from stem cell injury.

## Results

### Cdc42 is indispensable for enteroid survival.

**Figure 1:**
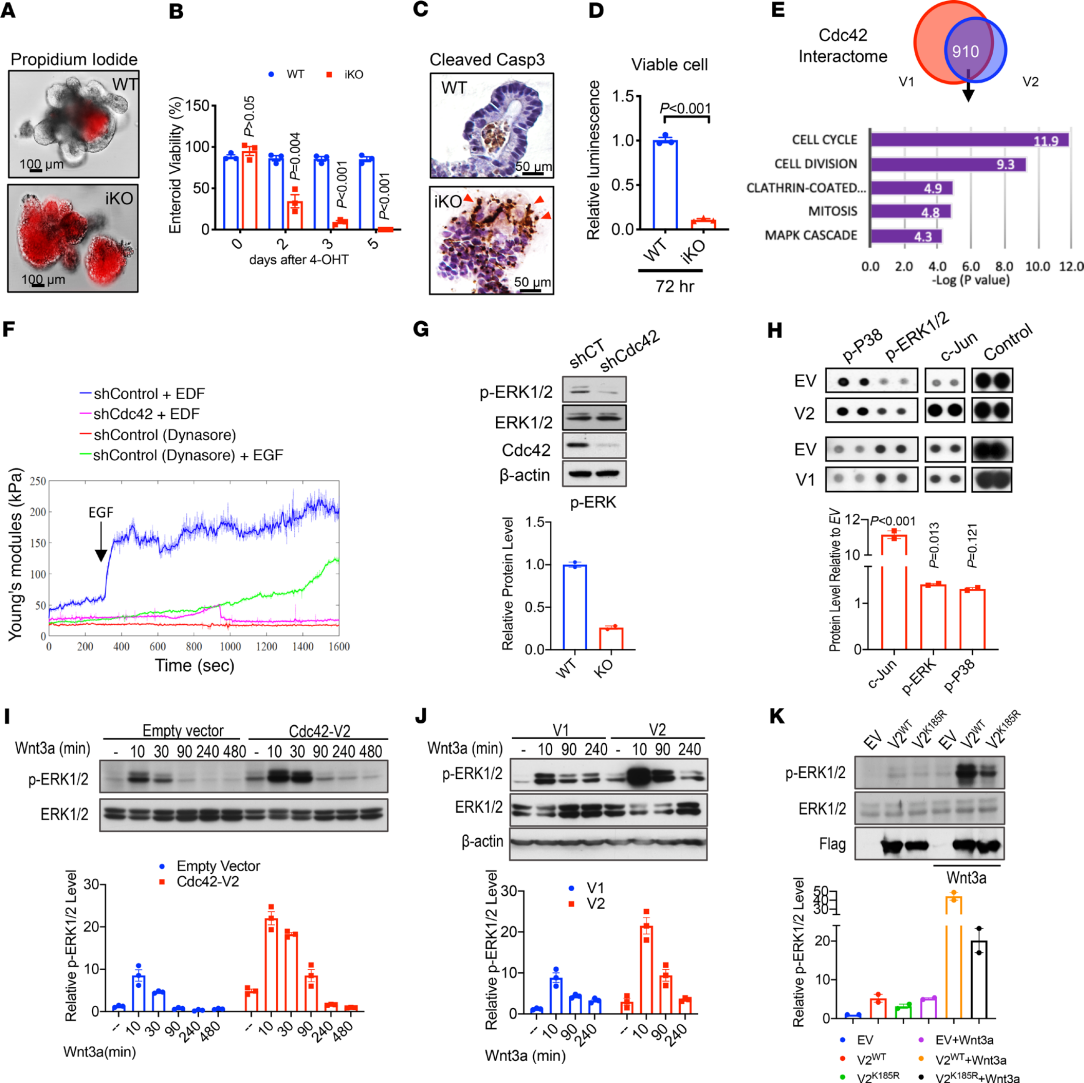
Enteroid survival requires Cdc42 for EGF/MAPK signaling. (**A**) Propidium iodide staining (red) showed massive cell death 72 hours after 4-OHT treatment of WT and *Cdc42^iKO^* enteroids. (**B**) After 4-OHT treatment of enteroids, viable enteroids were counted at days 2, 3, and 5 following treatment. Data represent 3 independent experiments, each containing 2 replicates per genotype. (**C**) WT and *Cdc42^iKO^* enteroid sections were stained for cleaved caspase-3 to detect apoptotic cells. Images shown represent 72 hours after treatment. (**D**) 3D-Glo luminescence assays were used to quantify cell viability in WT and *Cdc42^iKO^* enteroids 72 hours after 4-OHT treatment. Data represent 2 independent experiments, each containing 2 replicates per genotype. (**E**) HEK293 cells were transfected with Flag-tagged Cdc42-V1 or -V2. Mass spectrometry analysis of Flag-Cdc42 immunoprecipitates identified a variant-specific interactome. Venn diagram reveals 910 shared interactors. Functional annotation showed the top 5 most enriched clusters ranked by *P* value by DAVID Gene Functional Classification Tool. (**F**) Serum-starved Caco2 control and *Cdc42*-knockdown cells were analyzed by atomic force microscope. EGF was added to cells 300 seconds after recording of each sample. Young’s modulus (kPa) indicated EGF-induced nanomechanical changes on control Caco2 cell surface, with diminished responses in control cells preincubated with dynasore or in *Cdc42*-knockdown cells. Graphs represent 4 independent experiments. (**G**) Western blots for p-ERK1/2 were performed for control and stable *Cdc42*-knockdown Caco2 cells cultured in regular medium with no treatment. Data were quantified from 2 independent experiments. (**H**) Phospho-kinase array was used to search for functional pathways downstream of Cdc42. Among 43 kinase targets, overexpressing Cdc42 in HEK293 cells increased levels of p-ERK1/2, p38, and c-Jun, especially in V2-expressing cells compared with empty vector controls. Graph represents mean values from 2 replicates. (**I**) Western blots were used to determine change of p-ERK1/2 in serum-starved control and Cdc42-V2 expressing cells, following the addition of Wnt3a (100 ng/mL), in time course experiments. (**J**) Western blots for p-ERK1/2 were used to compare V1 versus V2’s MAPK-activating capabilities. (**K**) Mutant Cdc42-V2^K185R^ showed a reduced MAPK-activating capability compared with WT Cdc42-V2. Data were quantified from 2 independent experiments in **I**, **J**, and **K**. Please also see Supplemental Figure 1.

*Cdc42^ΔIEC^* mice with constitutive IEC-specific *Cdc42* ablation via *Villin-Cre* were viable (28). However, crypts isolated from *Cdc42^ΔIEC^* mice did not develop into enteroids in medium containing EGF, Noggin, and R-Spondin (ENR). Within 24 hours after seeding, all *Cdc42^ΔIEC^* crypts were growth arrested (Supplemental Figure 1A; supplemental material available online with this article; https://doi.org/10.1172/jci.insight.135923DS1). Because this growth deficit could be secondary to abnormal development by *Cdc42^ΔIEC^* intestinal epithelia, we used *Villin-CreER* to delete *Cdc42* from mature *Cdc42^fl/fl^ Villin-CreER* (*Cdc42^iKO^*) enteroids. Forty-eight hours after 4-hydroxytamoxifen (4-OHT) addition, the epithelial buds in over 60% of *Cdc42^iKO^* enteroids regressed, with cell death and cellular debris (Figure 1, A and B; and Supplemental Figure 1B). After 72 hours, 95% of *Cdc42^iKO^* enteroids accumulated significant amounts of propidium iodide, indicative of massive cell death (Figure 1B), which was further corroborated by elevated numbers of cleaved caspase-3–positive cells (Figure 1C) and a loss of over 90% live enteroid cells quantified by the CellTiter-Glo 3D Cell Viability Assay (Figure 1D). Viable enteroids were not detected at 5 days after *Cdc42* deletion, and the growth of *WT* (WT) enteroids was not affected by 4-OHT over this time period (Figure 1, A–D). Thus, maintenance of enteroid growth in ENR medium absolutely was dependent upon Cdc42 expression.

### Cdc42 is required for and sufficient to promote EGF/MAPK signaling ex vivo.

We first suspected a disrupted Wnt signaling in Cdc42-deficient enteroids; therefore, we tried to rescue the growth of Cdc42-deficient enteroids by enhancing canonical Wnt signaling via the glycogen synthase kinase-3β inhibitor CHIR99021. However, we failed to observe a reversing effect on death (Supplemental Figure 1C), suggesting that canonical Wnt pathway was not the primary pathway affected by Cdc42 deficiency. Treating serum-starved human Caco2 cells with EGF ligands (EGF and TGF-α), canonical Wnt ligand (Wnt3a), or Noggin activated Cdc42 GTPase activities within minutes (Supplemental Figure 1D), suggesting that multiple ISC growth factors could act through Cdc42 machinery.

These results led us to conduct an unbiased proteomic search for Cdc42-regulated survival signaling. Parallel mass spectrometry was done on canonical Cdc42 (V1) and its splicing variant (V2), both of which were 3xFlag-tagged and transfected into HEK293 cells. Proteomic analysis of Cdc42 V1 and V2 immunoprecipitates identified an overlapped interactome, where the 5 top functional clusters mapped to cell cycle, cell division, clathrin-coated endocytosis, mitosis, and MAPK cascade components (*P* < 0.001, Figure 1E and Supplemental Figure 1, E and F).

The identification of a clathrin and MAPK interactome components suggests that Cdc42 may control MAPK activation through clathrin-dependent endocytic EGFR signaling (37–41). EGF stimulates rapid receptor-mediated endocytosis that generates changes in plasma membrane elasticity measurable by atomic force microscope (42). Within 30 seconds of EGF loading, there was a robust increase in nanomechanical stiffness on the surface of control Caco2 cells (blue line, Figure 1F). This response was abolished in cells pretreated with the endocytosis inhibitor dynasore (green line, Figure 1F) (43). *Cdc42*-knockdown cells produced a much diminished (89% reduction in stiffness) and a delayed (by 10 minutes) response to EGF ligands (pink line, Figure 1F). This impaired nanomechanical response to EGF coincided with reduced intracellular phosphorylated ERK1/2 (p-ERK1/2) levels in *Cdc42*-knockdown cells (Figure 1G), illustrating Cdc42’s requirement for EGF-stimulated endocytosis.

To further dissect signaling cascades downstream of Cdc42, we examined 43 major intracellular kinase targets in HEK293 cells overexpressing either Cdc42 V1 or V2. Compared with cells expressing empty vector, phosphorylation levels of 3 major MAPK components, ERK1/2, c-Jun, and p38, were elevated in Cdc42-overexpressing cells, especially in V2-overexpressing cells (Figure 1H and Supplemental Figure 1G). We validated this finding in a time course experiment, in which overexpressing Cdc42 resulted in a 5-fold increase in p-ERK1/2 (V2 shown in Figure 1I). In addition to EGFs, canonical Wnt has also been shown to transactivate ERK (44). In the presence of Wnt3a, an ISC growth factor, Cdc42-V2 robustly elevated p-ERK1/2 (Figure 1, I and J). A side-by-side comparison of ERK-activating capabilities of the 2 variants showed that V2’s strength was 230% of V1’s (Figure 1J).

The stronger MAPK-activating capabilities by V2 were not due to different binding to the Cdc42 effector p21-activated kinase 1 (PAK1), which equally associated with both variants (Supplemental Figure 1H). Cdc42 V1 and V2 differ in the last 10 amino acids. Mutation of V2-specific lysine 185 (K185R) within the C-terminus poly-lysine region, reported to be essential for membrane attachment (45–47), led to a marked reduction in p-ERK1/2 levels (Figure 1K), suggesting that Cdc42’s MAPK-stimulating activity requires membrane association.

### Cdc42 is required for EGFR endocytosis.

**Figure 2:**
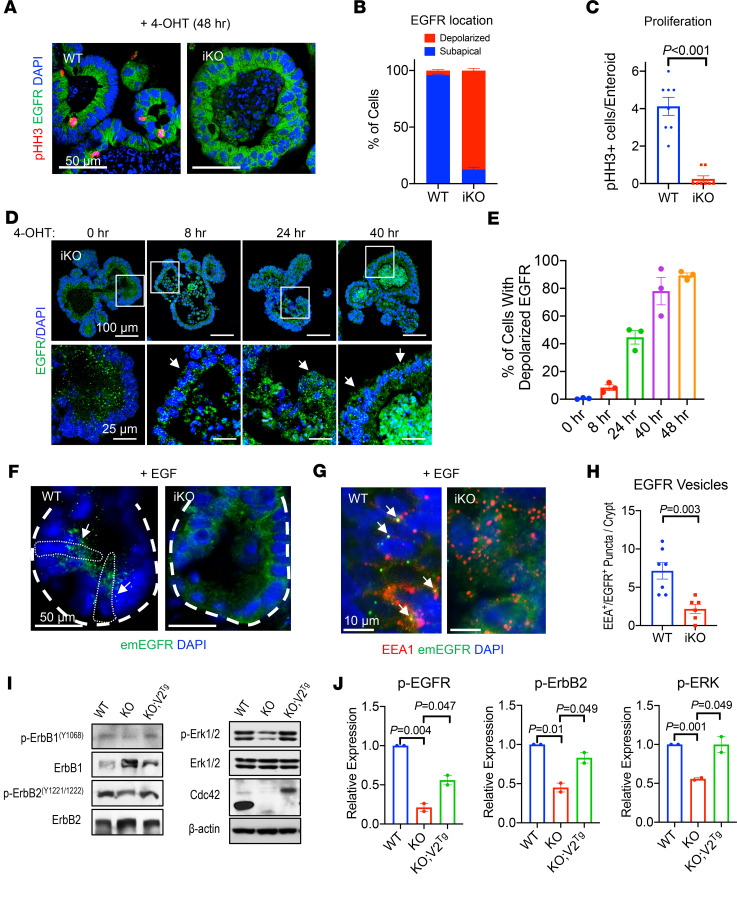
Cdc42 is indispensable for EGFR endocytosis. (**A**) WT and *Cdc42^iKO^* enteroids were treated with 4-OHT for 48 hours. Enteroid sections were stained for endogenous EGFR (green) and mitosis marker (pHH3, red). (**B**) Quantification of intracellular localization of EGFR in WT and *Cdc42^iKO^* enteroid cells, based on immunofluorescence staining of EGFR in 4-OHT–treated enteroids (48 hours). (**C**) pHH3^+^ cell number was quantified per enteroid. Data in **B** and **C** were quantified from 2 independent experiments. (**D**) Enteroid sections were stained for EGFR after 4-OHT treatment at 8, 24, and 40 hours. White arrows point to delocalized EGFR compared with EGFR localization in untreated enteroids. (**E**) Quantification of percentage of cells showing changed EGFR localization at indicated time points after 4-OHT addition. (**F**) Cdc42 mice were crossed to emEGFR mice to visualize EGFR. Thirty minutes after EGF injection, emEGFR vesicles (green, white arrows) were seen in Cdc42-WT mouse crypt cells but were barely detected in iKO crypt cells. White dotted circles indicate crypt base stem cells. (**G** and **H**) Double fluorescence analysis for EEA1 (red) and emEGFR (green, white arrows) detected endocytic EGFR in WT crypt cells but not *Cdc42^iKO^* cells 30 minutes after EGF injection. EEA1^+^EGFR^+^ puncta per crypt were quantified from 4 mice in 2 independent experiments. (**I** and **J**) Western blots for endogenous p-ErbB1 (Y1068), p-ErbB2 (Y1221/1222), and p-Erk1/2 were performed for WT, Cdc42-KO, and Cdc42 V2^Tg^ mouse intestines. Data represent 2 independent experiments. Please also see Supplemental Figure 2.

To examine the intrinsic impact of loss of Cdc42 on EGFR endocytic signalosome in the intestinal epithelium, *Cdc42* was inducibly knocked out in mature *Cdc42^iKO^* enteroids. Forty-eight hours following 4-OHT treatment, *Cdc42^iKO^* enteroid cells showed a noticeable loss of polarized intracellular EGFR distribution (Figure 2, A and B) and a reduction in phosphohistone H3–positive (pHH3^+^) mitotic activity (Figure 2, A and C). We performed a time course experiment to determine whether EGFR delocalization preceded enteroid cell death. Twenty-four hours after 4-OHT addition, 45% of enteroid cells exhibited altered localization of EGFR (Figure 2, D and E), while apoptotic cells remained rare (below 5%, Supplemental Figure 2, A and B). At 40 hours, approximately 80% of enteroid cells showed EGFR delocalization, but the vast majority of apoptotic events only occurred after 40 hours and peaked at 72 hours (Supplemental Figure 2, A and B). These data suggested that EGFR abnormality occurred before enteroid cell death, suggesting a possible link between EGFR abnormality and survival.

To directly observe EGFR trafficking in vivo, *Cdc42^iKO^* mice were crossed to a CRISPR/Cas9-engineered emerald-EGFR (emEGFR) mouse allele (48). Administration of a pulse of EGF to WT mice elicited a marked induction, within 30 minutes, of emEGFR vesicles as well as emEGFR^+^ and early endosome antigen–positive (EEA^+^) vesicles (Figure 2, F and G), which were diminished in *Cdc42^iKO^* crypt cells (Figure 2H). This reduction of EGF-stimulated endocytic signalosomes in *Cdc42^iKO^* crypt cells echoed significant decreases in levels of p-ErbB1 (Y1068) and p-ErbB2 (Y1221/1222), active forms of major EGFRs in *Cdc42^iKO^* IECs (Figure 2, I and J). Of note, total levels of both receptors were increased, and there were residual p-ErbB1, p-ErbB2, and p-Erk1/2 in *Cdc42^iKO^* IECs (Figure 2, I and J). These data suggested a changed EGFR compartmentalization and a possibly affected MAPK activation in *Cdc42^iKO^* IECs in vivo.

### Cdc42 engages EGFR and endocytosis machinery.

**Figure 3:**
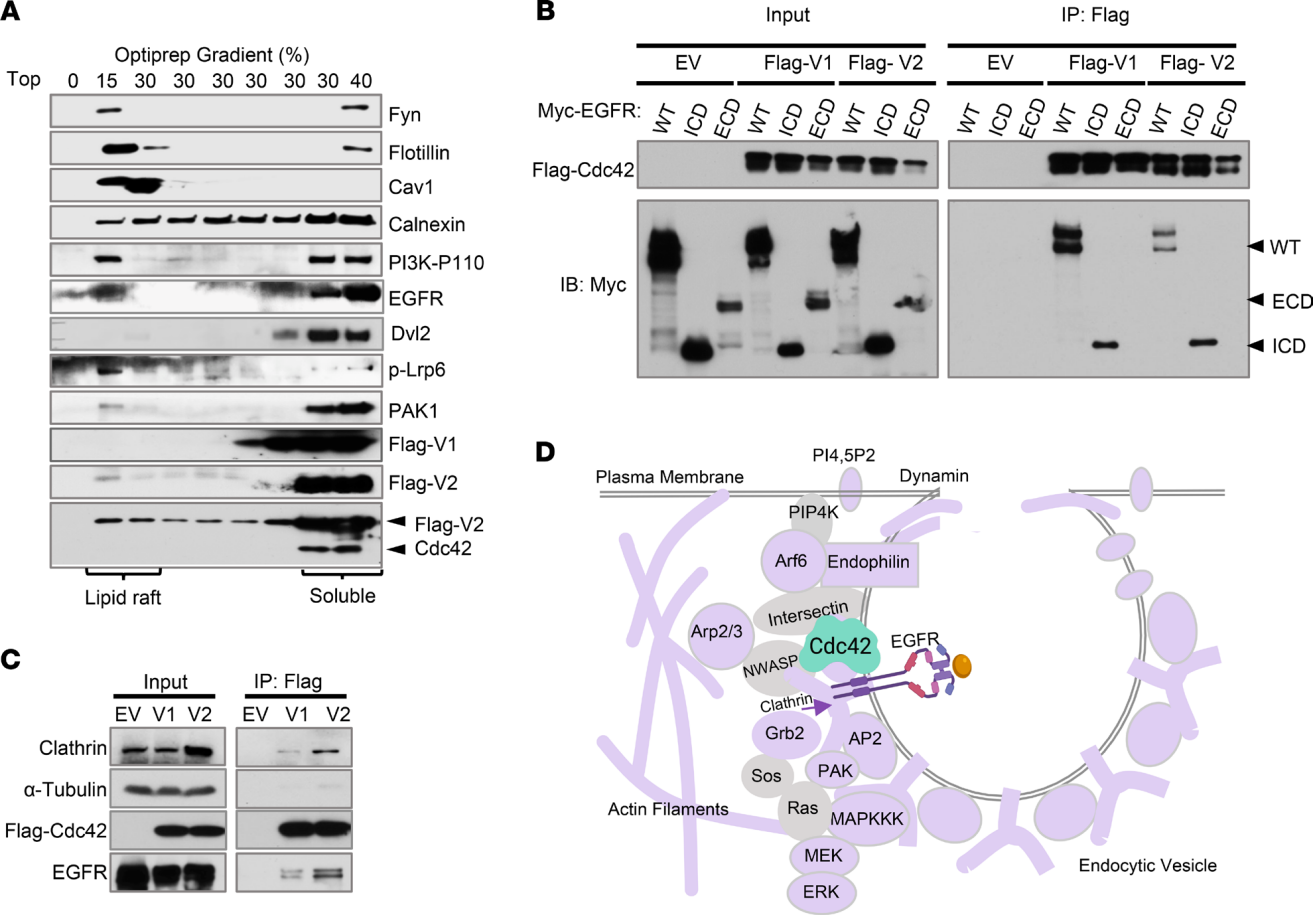
Cdc42 engages EGFR and endocytosis machinery. (**A**) OptiPrep gradient cellular fractionation of HEK293 cells showed cosedimentation of Cdc42-V2 with EGFR, p-LRP6, and PI3K-P110 in lipid raft fractions marked Fyn, Cav1, and Flotillin. Calnexin is an ER marker. Data represent 3 independent experiments. (**B**) Coimmunoprecipitation analysis showed that Flag-tagged Cdc42 associated with full-length EGFR and EGFR intracellular domain (ICD) but not the extracellular domain (ECD) in HEK293 cells. (**C**) Coimmunoprecipitation analysis showed that, compared with V1, V2 exhibited a stronger interaction with endogenous EGFR and clathrin in HEK293 cells. (**D**) A diagram illustrating the structural and functional protein components found in Cdc42 proteomics that were known to regulate clathrin-mediated trafficking, EGFR endocytosis, and MAPK cascade.

Ligand-stimulated EGFRs are associated with lipid rafts that are internalized to transduce MAPK signaling (49–53). When lipid rafts were extracted from V2-overexpressing cells using OptiPrep gradient cellular fractionation (54), a pool of V2 cosedimented with the lipid raft proteins Flotillin (55) and Caveolin-1 (56), along with EGFR, tyrosine receptor Fyn (57), phosphoinositide 3 kinases (PI3Ks) (58), and Wnt-activated pLrp6 (59, 60) (Figure 3A).

Coimmunoprecipitation analysis indicated that both V1 and V2 associated with EGFR through binding to the receptor’s intracellular domain (ICD) (Figure 3B, IP Flag panel), and this was further validated for endogenous EGFR and Cdc42 variants (Figure 3C). Compared with Cdc42 V1, the V2 variant exhibited a stronger association toward both EGFR and clathrin (Figure 3C), consistent with V2’s demonstrated stronger MAPK-activating capability (Figure 1J). A further examination of Cdc42 interactomes identified numerous structural and signaling components, including actin, IQGAP1/2/3, clathrin, AP-2, Arp2/3, MAPK kinase kinase 7 (MAPKKK7), p38, PAK4, and so on (Supplemental Table 1 and Supplemental Figure 2C) reported to be involved in EGFR endocytosis and MAPK cascade (61–65) (Figure 3D). Thus, Cdc42 directly engaged EGFR endocytosis machinery for MAPK signaling.

### IEC overexpression of Cdc42-V2 alters the epithelial differentiation program.

**Figure 4:**
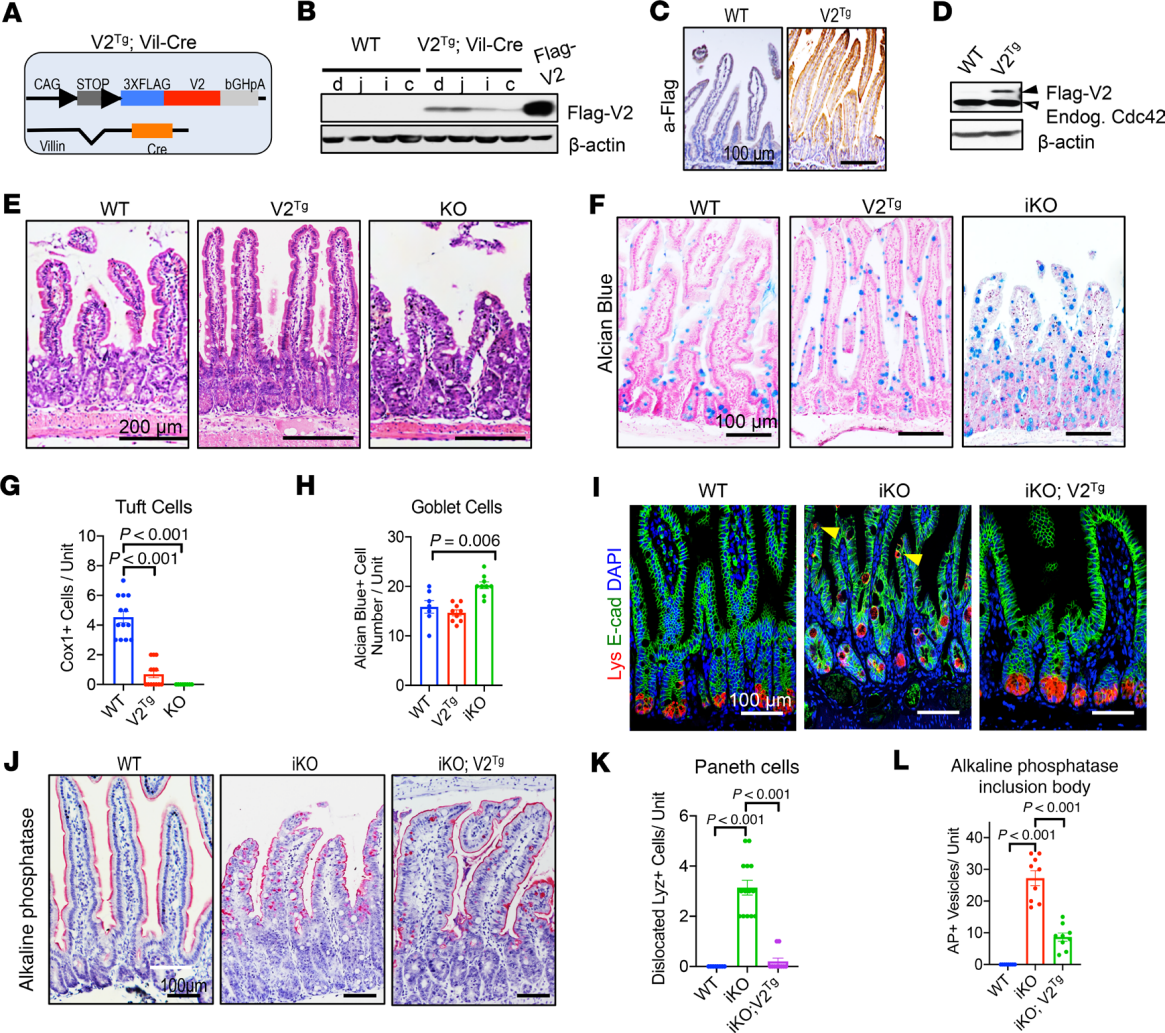
Overexpressing Cdc42 V2 in mouse intestinal epithelium affects differentiation. (**A**) A schematic diagram showing the strategy of developing a Cdc42 V2^Tg^ mouse allele, which conditionally expressed a Flag-tagged Cdc42-V2 in a Cre-dependent manner. A loxP-stop-loxP-3xFlag-V2-bGHpoly(A) cassette was inserted downstream of a chick actin (CAG) promoter. (**B**) Western blots for Flag detected Flag-V2 expression in duodenum, jejunum, ileum, and colon of V2^Tg^ driven by *Vil-Cre*. HEK293 cells expressing Flag-V2 were used as positive controls. (**C**) Immunohistochemistry for Flag detected Flag-V2 expression in V2^Tg^ mouse IECs. Images are representative of 3 independent mice per genotype. (**D**) Western blots for Cdc42 showed that the transgenic expression of V2 (upper band) in a V2^Tg^ line was approximately 40% of endogenous Cdc42 (empty arrowhead). (**E**) H&E staining of mouse intestines. V2^Tg^ small intestines showed longer villi and more crypts; KO showed blunted villi. (**F**) Alcian blue staining of mouse intestinal sections of indicated genotypes. (**G**) Quantification of cyclooxygenases 1–positive (Cox1^+^) cell number per crypt-villus unit. (**H**) Quantification of Alcian blue–positive cells per crypt-villus unit. (**I**) Immunofluorescence staining for lysozyme (red), E-cad (green), and DAPI (blue). (**J**) Alkaline phosphatase (AP) staining showed representative AP^+^ inclusion bodies in KO IECs. Scale bar: 100 μm. (**K**) Quantification of mislocalized Paneth cells. (**L**) Quantification of AP^+^ inclusion bodies. Data in **G**, **H**, **K**, and **L** were quantified from multiple intestinal sections of a total of 3 animals per genotype. Please also see Supplemental Figures 3 and 4.

To date, study of Cdc42 has been hampered by the absence of a specific gain-of-function in vivo model. We found that, in contrast to the ubiquitous expression of Cdc42-V1, expression of V2 could be detected in mouse enteroids (Supplemental Figure 3A) and fetal intestines, while its level was reduced in adult intestines (Supplemental Figure 3B). Based on V2’s stronger MAPK-activating capacity, we sought to determine whether elevating Cdc42 activity in adult IECs by overexpressing V2 (a naturally expressed protein) might enhance epithelial survival in vivo. We thus established an inducible mouse allele (referred to as V2^Tg^), which allowed a Cre-activated, cell-specific production of a Flag-tagged Cdc42-V2 (Figure 4A).

We validated IEC-specific Cdc42-V2 production in small intestinal and colonic epithelia of independent V2^Tg^ founders crossed to *Villin-Cre* or *Villin-CreER* drivers by Western blot (Figure 4B) and by immunohistochemistry (Figure 4C). A V2^Tg^ founder producing V2 at approximately 40% of endogenous Cdc42 was selected for further experiments (Figure 4D). *Villin-Cre V2^Tg^* intestines had longer villi and more crypts (Figure 4E and Supplemental Figure 4, A and B). Examination of intestinal epithelial cell types revealed that V2^Tg^ intestine had a reduction of Tuft cells by 80% (Figure 4G and Supplemental Figure 4, C–E) but had no change in goblet cells (Figure 4, F and H). In contrast, *Cdc42^ΔIEC^* intestines showed a complete loss of Tuft cells and a 30% increase in goblet cells (Figure 4G and Supplemental Figure 4, C–E).

Interestingly, expressing *V2^Tg^* in *Cdc42^ΔIEC^* intestinal epithelium significantly mitigated the Paneth cell defect (Figure 4, I and K) and microvillus inclusion phenotype (Figure 4, J and L; and Supplemental Figure 4F) previously reported in *Cdc42^ΔIEC^* intestines (28). These data functionally validated this newly developed *V2^Tg^* mouse allele and revealed an unknown impact of Cdc42 on Tuft cell differentiation.

### Overexpressing Cdc42-V2 robustly elevates ISC function in vivo.

**Figure 5:**
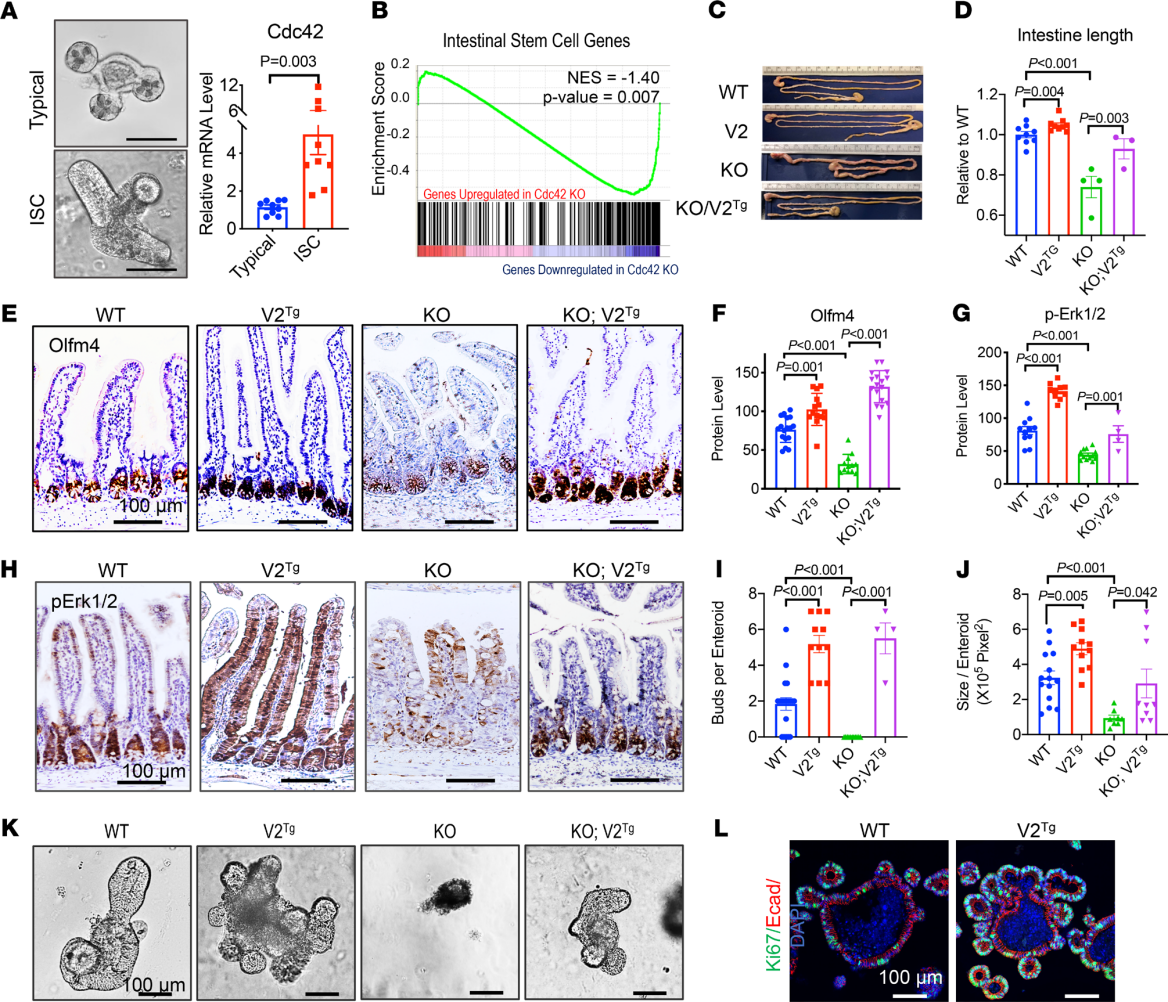
Overexpressing Cdc42-V2 enhances ISC function and MAPK signaling. (**A**) Images of a typical enteroid and an ISC-enriched enteroid induced by CHIR99021 and valproic acid. Quantification RT-PCR for Cdc42 detected higher mRNA level in ISC enteroids. Scale bar: 100 μm. (**B**) Gene set enrichment analysis (GSEA) of bulk RNA-Seq showed a significantly reduced Lgr5 ISC gene signature (*P* = 0.007) in *Cdc42^iKO^* compared with WT IECs. (**C** and **D**) Representative gross morphology of WT, V2^Tg^, KO, and KO V2^Tg^ mouse intestines. Note V2^Tg^ intestine was noticeably longer than littermate WT. (**E** and **F**) Immunohistochemistry for Olfm4 showed increased crypt base ISCs in V2^Tg^ intestines and decreased crypt base ISCs in KO intestines. Olfm4 protein level within crypts was quantified from multiple intestinal sections of a total of 3 animals per genotype. (**G** and **H**) Compared with WT mice, p-Erk1/2^+^ cells were expanded in V2^Tg^ intestines and became scattered in KO intestines. p-Erk1/2 protein level within a crypt-villus unit was quantified from multiple intestinal sections of a total of 3 animals per genotype. (**I** and **J**) The average number of epithelial buds per enteroid and the average size of enteroids was quantified from 3 animals per genotype. (**K**) Representative enteroids of designated genotypes. (**L**) Immunofluorescence staining of enteroid sections showed increased Ki-67^+^ cells in epithelial buds of V2^Tg^ enteroids. Please also see Supplemental Figure 5.

Inhibition of MAPK by EGFR inhibitor in enteroid culture was shown to promote Tuft cell differentiation (3). The observed loss of Tuft cell differentiation in both gain and loss of Cdc42 function prompted us to examine the impact of Cdc42 on MAPK activity and ISC function. Quantitative real-time PCR (RT-PCR) detected a 5-fold increase in *Cdc42* mRNA level in ISC-enriched enteroids treated with CHIR99021 and valproic acid (66, 67) as compared with enteroids typically grown in ENR medium (Figure 5A). Bulk RNA-Seq of *Cdc42^ΔIEC^* intestines showed a transcriptomic reduction of leucine rich repeat containing G protein coupled receptor 5 (Lgr5) ISC gene signature (*P* = 0.007, Figure 5B).

*Cdc42^ΔIEC^* mice had shorter intestines (73% of WT littermates, Figure 5, C and D), while *Villin-Cre V2^Tg^* mice with elevated Cdc42-V2 in IECs had longer intestines, 112% of WT mice (Figure 5, C and D), indicative of enhanced ISC function (68, 69). *V2^Tg^* intestines had increased olfactomedin 4 (Olfm4) levels in crypts (Figure 5, E and F), and elevated gene signatures of both fast-cycling and “quiescent” ISCs, in particular Bmi1 (Supplemental Figure 5A). Importantly, a pronounced expansion of p-Erk1/2^+^ active cell population to the villus region was detected in *V2^Tg^* intestines (Figure 5, G and H). By contrast, p-Erk1/2^+^ cells in WT mice were restricted to crypt and transit-amplifying compartments as previously reported (70, 71). Scattered p-Erk1/2^+^ cells were present throughout *Cdc42^ΔIEC^* villus epithelium. We also detected elevated β-catenin levels in *V2^Tg^* intestines (Supplemental Figure 5, B and C), in overall agreement with an enhanced ISC function in homeostasis.

We established *Cdc42^ΔIEC^ V2^Tg^* mice where deletion of endogenous Cdc42 and production of the V2 transgene were driven by Cre in the same IECs. *Cdc42^ΔIEC^* V2^Tg^ mice showed largely restored IEC phenotypes compared with *Cdc42^ΔIEC^* mice (Figure 5, C–H). These phenotypic restorations were accompanied by restoration of p-ErbB1, p-ErbB2, and p-Erk1/2 levels in *Cdc42^ΔIEC^* V2^Tg^ mouse intestines (Figure 2I). Ex vivo, V2^Tg^ enteroids exhibited a much more robust epithelial budding (Figure 5, I and K), larger sizes (Figure 5, J and K), and increased proliferation activities (Figure 5L). Thus, elevating epithelial Cdc42-V2 autonomously enhanced ISC function at steady state.

### Cdc42-V2 mitigates injury-induced epithelial damage.

**Figure 6:**
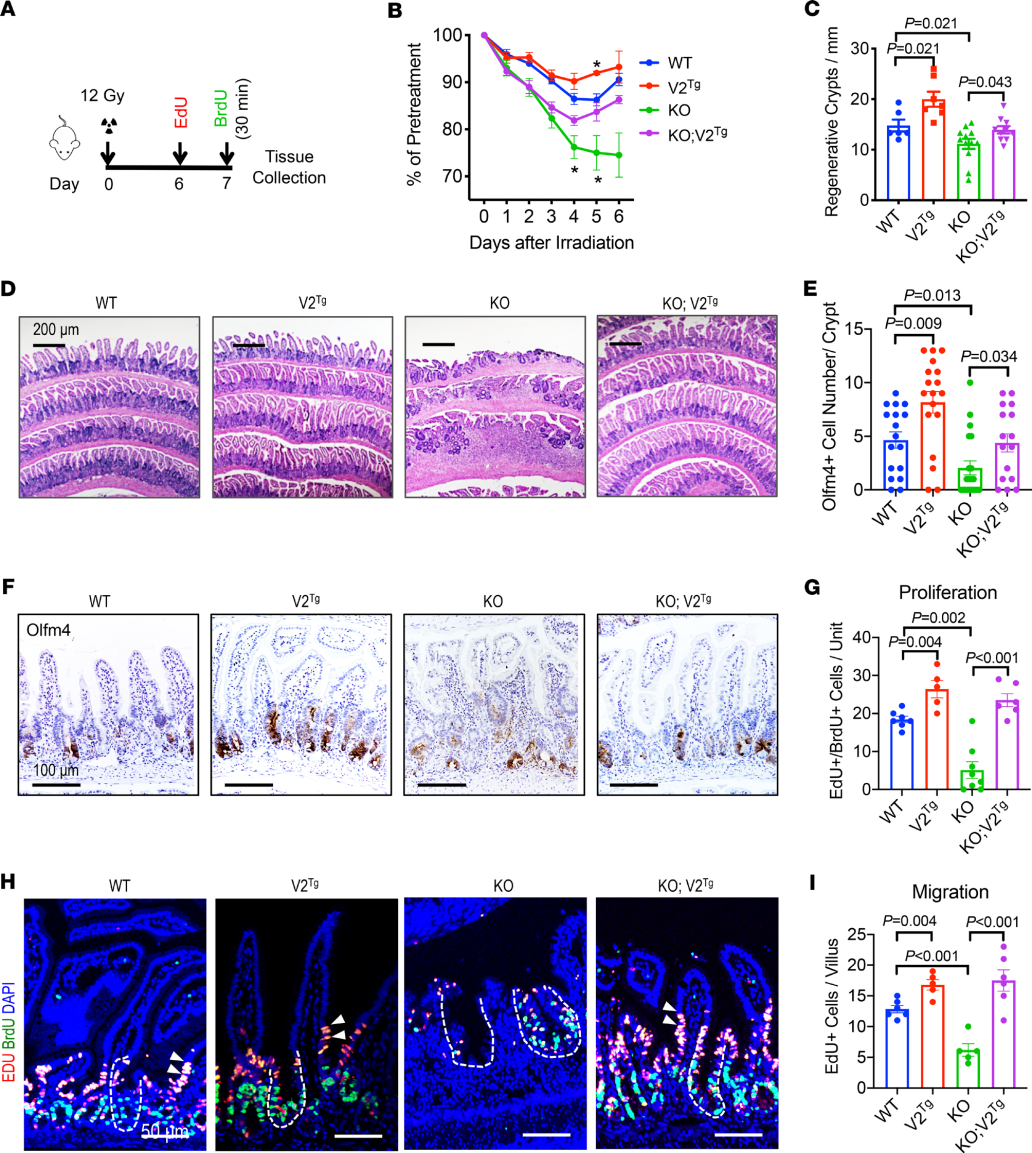
Elevating the epithelial Cdc42-MAPK program mitigates mucosal damage following ISC loss. (**A**) Mice received a 12 Gy total-body irradiation and were sacrificed 7 days after irradiation. Cycling IECs were labeled by sequential injection of EdU (1 day) and BrdU (30 minutes) before sacrifice. (**B**) Percentage of body weight changes were presented as averages of each genotype group (*N* = 8 per genotype). Note there was death of KO animals during the experiment following irradiation. Asterisks indicate *P* < 0.05 compared with WT mice. (**C**) Average numbers of regenerative crypts per millimeter were counted from multiple ileal sections of 3 postirradiation animals per genotype. (**D**) H&E-stained postirradiation mouse ileal sections of various genotypes 3 days after irradiation. (**E** and **F**) Olfm4^+^ cells were counted from immunohistochemistry performed on multiple ileal sections of 3 postirradiation animals per genotype. Representative images show Olfm4 staining of different genotypes 3 days after irradiation. (**G** and **H**) EdU (red) and BrdU (green) were stained. Average numbers of EdU- and BrdU-labeled IECs per crypt-villus unit were quantified from multiple sections of 3 postirradiation animals per genotype. Dashed lines indicate crypts; white arrows point to EdU^+^ and BrdU^+^ IECs migrating to villi. Nucleus was counterstained with DAPI (blue). Scale bar: 50 μm. (**I**) Average numbers of only EdU-labeled IECs in upper crypt and villus region were quantified from multiple sections of 3 postirradiation animals per genotype. Please also see Supplemental Figure 6.

Prompted by above results, the impact of V2 expression on epithelial regeneration after injury was examined. ISCs are sensitive to irradiation (72), with fast-cycling ISCs dying within 2 days after irradiation (73–75). Mice were exposed to 12 Gy total-body irradiation (Figure 6A). Compared with WT littermates, Cdc42*^ΔIEC^* mice lost nearly 25% of body weight 4 days after irradiation (green line, Figure 6B). This was accompanied by declining body conditions and 25% postirradiation mortality of Cdc42*^ΔIEC^* mice within a week. All *WT*, *V2^Tg^*, and *Cdc42^ΔIEC^ V2^Tg^* mice survived the experimental duration. *V2^Tg^* mice were resistant to body weight loss and exhibited an earlier weight recovery (red line, Figure 6B), while *Cdc42^ΔIEC^ V2^Tg^* mice showed improved body weight and health conditions compared with *Cdc42^ΔIEC^* mice (purple line, Figure 6B). Examination of intestines revealed that *Cdc42^ΔIEC^* intestines were 24% shorter than WTs (Supplemental Figure 6, A and B), with reduced numbers of regenerative crypts (Figure 6, C and D; and Supplemental Figure 6C). Consistent with the elevation of stem cell marker mRNA level, more Olfm4^+^ cells were detected in each crypt of the V2^Tg^ mice (Figure 6, E and F). Likewise, there were overall more Olfm4^+^ crypts in V2^Tg^ mice than in WT mice (Supplemental Figure 6, D and E). Furthermore, 24-hour EdU labeling (red) followed by a 30-minute BrdU labeling (green) before sacrifice (Figure 6A) indicated that *Cdc42^ΔIEC^* mice had reduced cycling cells as well as cell cycle reentry indicated by EdU^+^BrdU^+^ cells (Figure 6, G and H). *Cdc42^ΔIEC^* mice also had reduced numbers of cells migrating into villus epithelia (arrows in Figure 6, H and I). Cdc42-V2 overexpression enhanced IEC proliferation and migration and restored above-illustrated phenotypic defects in *Cdc42^ΔIEC^ V2^Tg^* mice (Figure 6, B–I; and Supplemental Figure 6, A–E).

### Overexpressing Cdc42-V2 in ISCs enhances epithelial lineage tracing after irradiation injury.

**Figure 7:**
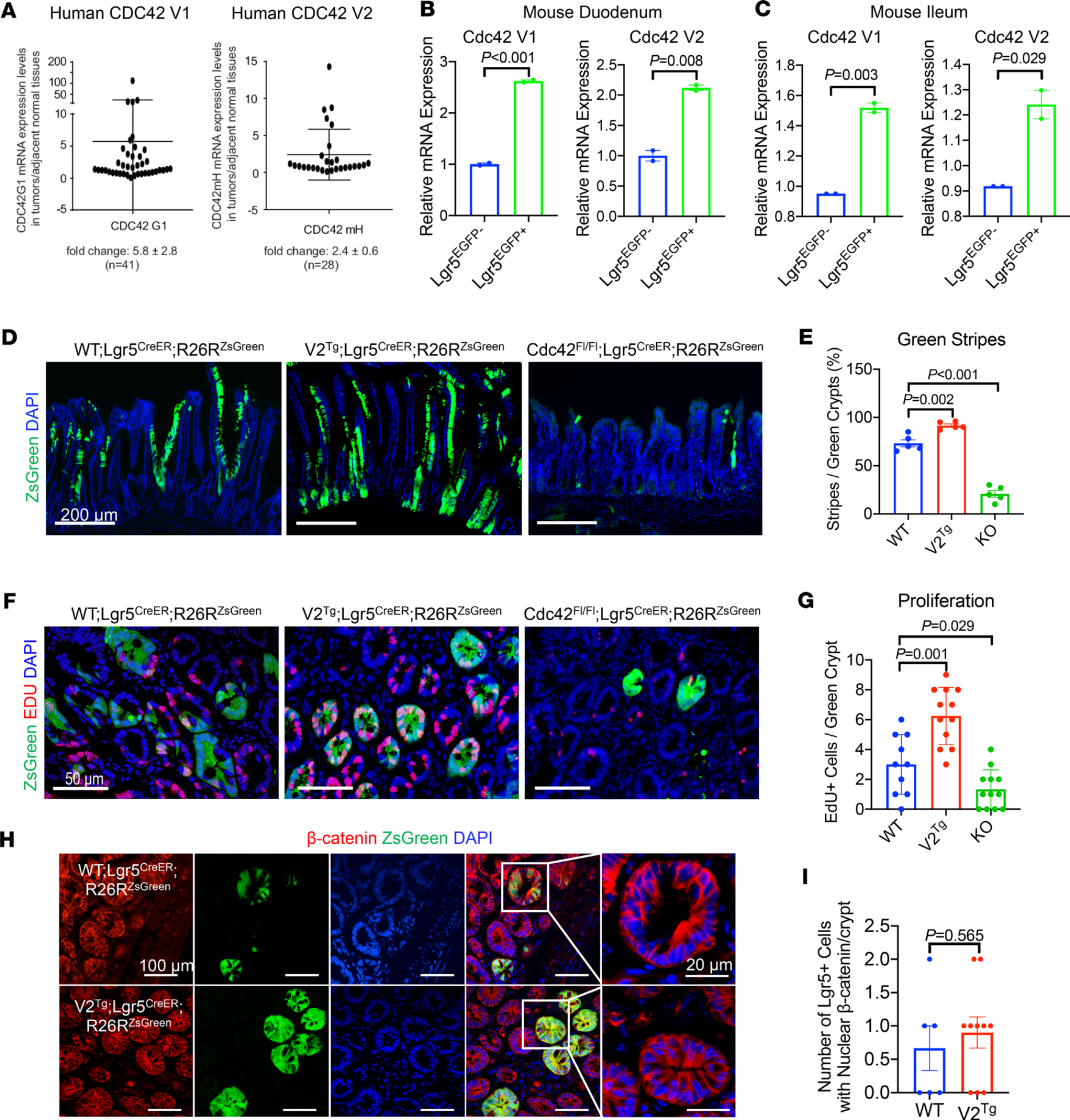
Elevating Cdc42 in ISCs promotes injury-induced regeneration. (**A**) TaqMan RT-PCR for human CDC42-V1 and -V2 mRNA was performed on colon tumor tissues and adjacent nontumor tissues. Data were graphed as ratio of tumor over adjacent tissue. Elevated CDC42-V1 was shown in 27 out of 41 tumors while 12 out of 28 tumors had elevated CDC42-V2. (**B**) Quantitative PCR for mouse Cdc42 variants was performed on FACS-sorted Lgr5^EGFP+^ versus Lgr5^EGFP–^ IECs from WT mouse duodenums. (**C**) Quantitative PCR for mouse Cdc42 variants was performed on FACS-sorted Lgr5^EGFP+^ versus Lgr5^EGFP–^ IECs from WT mouse ileums. (**D** and **E**) Lineage tracing of Lgr5 ISCs of distinct (*Cdc42-WT*, *V2^Tg^*, or *KO*) genotypes 7 days after irradiation using an R26R^ZsGreen^ reporter. EdU was injected 6 hours before sacrifice to identify cycling ISC descendants. Lineage tracing events were presented as percentage of observed green stripes out of total number of green crypts within the same field. Data represent multiple sections from 2 postirradiation animals. Note that V2^Tg^ and KO ISCs showed increased and diminished lineage tracing events. (**F** and **G**) The average numbers of EdU^+^ cells per green crypt were quantified from multiple sections of 2 postirradiation animals. (**H** and **I**) The average numbers of nuclear β-catenin^+^ cells per green crypt were quantified from multiple sections of 2 postirradiated *Lgr5^CreER-IRES-EGFP^ R26R^zsGreen^* *Cdc42-WT* and *Lgr5^CreER-IRES-EGFP^ R26R^zsGreen^ V2^Tg^* mice.

Quantitative PCR showed significantly elevated expression of canonical CDC42 (V1) in human colorectal cancer tissues, compared with adjacent normal tissues (*N* = 41 pairs of samples; Figure 7A), consistent with a previous report (76). Using a CDC42-V2–specific TaqMan probe, we detected an average of 2.5-fold increase in cancer tissues compared with adjacent normal tissues (*N* = 28 pairs of samples; Figure 7A). In addition, Lgr5^EGFP+^ ISCs FACS-sorted from WT Lgr5^CreER-IRES-EGFP^ mouse duodenum and ileum showed elevated expression levels of both Cdc42 variants when compared with sorted Lgr5^EGFP–^ epithelial cells (Figure 7, B and C). We concluded that although Cdc42 is ubiquitously expressed in all epithelial cells, both variants are preferentially elevated in proliferating cells.

As compared with WT mice, *V2^Tg^* mice exhibited a resistance to irradiation-induced loss of Olfm4^+^ cells (Figure 6, G–I); this suggested that a higher level of Cdc42-V2 might favor ISC survival and regeneration. Pan-IEC Cre drivers, such as Villin-Cre, provide limited insights into the role of Cdc42 specific to ISCs. We specifically overexpressed V2^Tg^ in Lgr5 ISCs by deriving *Lgr5^CreER-IRES-EGFP^ R26R^zsGreen^ V2^Tg^* mice. Lineage tracing of Lgr5^+^ ISCs was conducted 3 days after 12 Gy irradiation of *Lgr5^CreER-IRES-EGFP^ R26R^zsGreen^ V2^Tg^* mice, where V2 overproduction was initiated from Lgr5 ISCs. Compared with Cdc42-WT mice, *Lgr5^CreER-IRES-EGFP^ R26R^zsGreen^ V2^Tg^* mice showed significantly more lineage tracing events (illustrated by ratio of green stripes to green crypts, Figure 7, D and E), which were accompanied by elevated mitotic cells in traced crypts (Figure 7, F and G). Nonetheless, ISCs overexpressing V2^Tg^ did not appear to have elevated levels of nuclear β-catenin (Figure 7, H and I), again suggesting that the enhanced epithelial regeneration was not directly related to canonical Wnt signaling.

In contrast, *Cdc42^fl/fl^ Lgr5^CreER-IRES-EGFP^ R26R^zsGreen^* mice lacking Cdc42 in ISCs showed drastically reduced lineage tracing events after irradiation (Figure 7, D and E) accompanied by reduced mitosis (Figure 7, F and G). Taken together, our data suggested that Cdc42 is an epithelial intrinsic machinery essential for mucosal repair in response to injury-induced damage.

## Discussion

This report provides molecular, genetic, and cell biological insight into the underlying mechanism of the extraordinary regenerative capacity of intestinal epithelium in response to injury. Our data shed light on how exactly Cdc42 potentiates MAPK pathway and possibly other signaling pathways related to ISC survival. Based on atomic force microscopic study, proteomics, and kinase array analysis, we showed that Cdc42 is required for ligand-stimulated EGFR endocytosis and MAPK signaling. Cdc42-deficient IECs lost responses to both EGF and Wnt ligands that activate MAPK.

Rho family GTPases (Rho, Cdc42, and Rac) share a set of guanine nucleotide exchange factor, GTPase-activating protein, guanosine diphosphate dissociation inhibitor, and downstream effector proteins and are involved in regulating common signaling transduction pathways (77–79). Cdc42 sometimes exhibits distinct functions from the other 2 Rho GTPase members (80, 81). Notably, Cdc42 inhibits activities of Rho, for example, in the regulation of epithelial cell apical tension (82) or during activation of Rhotekin in optic tectal cells (83). Regarding EGFR/MAPK signaling, in vitro studies showed that Rac is activated by EGF during colonic epithelial migration (84) and during MAPK-mediated Cox2 production (85). Thus, Rac might be playing a role downstream of EGF/MAPK cascade in the intestinal epithelium. This might partially explain the fact that no compensatory mechanism ex vivo rescued the death of Cdc42-deficient enteroids.

Multiple gain- and loss-of-function experimental platforms in our study consistently supported an impact of Cdc42 on the EGFR/MAPK pathway. EGFR signaling possesses a foundationally conserved role in regulating gut development and cell proliferation in lower (86–92) and higher eukaryotic species (93, 94). The requirement of EGF-initiated signaling for epithelial survival and renewal is extensively documented (48, 95–98) and is proposed to keep Lgr5^+^ ISCs in a constitutively active state (3). EGFR/MAPK signaling may enhance intestinal epithelial survival by preventing cell shedding (99). Proper intraepithelial EGFR localization has also been shown to define cell type–specific functions (100). These studies and the reported contribution of EGFR signaling to injury-induced tissue regeneration (101, 102) were consistent with our observed impact of Cdc42 on EGFR/MAPK cascade and cell survival in vitro and in vivo.

Within the lipid raft compartment, Cdc42 was also cosedimented with Wnt receptor and PI3K. Thus, Cdc42 must also crosstalk with other signaling components that use endocytic machinery, such as the Wnt and PI3K cascades. Cdc42 loss or gain of function was likely to exert pleiotropic influences on other non-EGF survival pathways. Nevertheless, our data revealed a more consistent impact of Cdc42 on MAPK signaling than its impact on other pathways. Both Wnt and EGF are ISC niche factors, and both have been documented to activate the MAPK pathway (103). For example, Wnt3a activates JNK (104), p38 (62), and ERK (105). Wnt3a could directly activate ERK via Raf or through AP1 and TCF (105). Future studies will be necessary to delineate the collateral effects of Cdc42 on each individual signaling pathway.

Although our analysis heavily focused on cell survival signaling, given Cdc42’s documented roles in controlling cell polarity and cytoskeletal organization, it is certain that the general Cdc42-dependent structural regulations are closely associated with cellular responses to external growth signals. Cdc42 could control apical and basolateral deployment of membrane proteins, such as EGFR, to facilitate signal receiving. Cdc42 may also control epithelial junctional integrity, thereby affecting Hippo signaling. Furthermore, the observed Paneth cell defects suggested an impact of Cdc42 on the Ephrin-EphB3 signaling shown to regulate Paneth cell positioning at crypts (106). Finally, the formation of microvillus inclusion in Cdc42-deficient enterocytes reflected a robust dysregulation of cortical actin in these cells. Thus, Cdc42-dependent cellular structural regulation must be strongly coupled to its impact on intracellular survival signaling activities.

Cdc42 has 2 physiologically transcribed isoforms with one being universally expressed (V1) while the other (V2) was initially found to be enriched in neuronal tissues. The major distinction between them was a selective splicing into a distinct last exon that encodes for the very C-terminal 10 amino acids. While both variants can be prenylated, only V2 can be palmitoylated at a unique C-terminal cysteine residue that was absent from the conventional Cdc42 (V1) (33). Studies in neuronal cells suggested Cdc42-V2’s stronger incorporation into lipid rafts (32, 33, 35, 107–109), which was proposed as a potential mechanism for its greater traffic and concentration in the dendritic spines (32, 34). It was postulated that Cdc42-V2 acted to promote the formation of subcellular postsynaptic structures, leading to a greater synaptic plasticity during brain activity (35), whereas the canonical Cdc42 aided axonogenesis (31, 36). Our data suggested that the stronger MAPK-activating capability by Cdc42-V2 was likely mediated by its enhanced affiliation with lipid compartments.

The newly engineered Cdc42 V2^Tg^ mice allowed a direct demonstration of a robust impact of Cdc42 gain of function on MAPK activation and ISC regeneration. Cdc42-V2 is physiologically expressed in enteroids and intestinal epithelium at lower levels compared with canonical Cdc42. Cdc42 V2^Tg^ mice provided a tool to enhance Cdc42 function. Findings reported here might represent the first step toward the possibility of enhancing ISC function by elevating Cdc42 activity, potentially through promoting the alternative splicing of Cdc42 variant 2 via regulators that have been reported (110). Our observed alterations in intestinal epithelial differentiation in Cdc42 loss- and gain-of-function models added to the reported impact of MAPK on the differentiation of intestinal epithelial cell types, including Paneth cells, goblet cells, and Tuft cells (3, 111, 112). Taken together, the cell-autonomous effects of Cdc42-V2 on epithelial regeneration have important implications for mitigating gastrointestinal pathologies that require an enhanced tissue repair program.

## Methods

### Mice.

The Cdc42 flox (113), emEGFR (48), Villin-Cre (114), Villin-CreER (115), Lgr5^EGFP-IRES-CreER^ (116), and Rosa26R-ZsGreen mice (117) have been previously described. A conditional *Cdc42-V2^Tg^* mouse allele was established by inserting the 3′Flag-Cdc42-V2 coding sequence downstream of a CAG promoter and a lox-CAT-lox cassette, at the unique BamH1 restriction site, followed by a 225 bp bovine growth hormone poly(A) sequence that does not contain coding sequence. All mice were maintained on a 12-hour light/12-hour dark cycle and provided with food and water ad libitum in individually ventilated cages under specific pathogen–free conditions at Rutgers University animal facility. Experimental comparisons were strictly made among littermates. Key resources used in this study can be found in Supplemental Table 2.

### Total-body gamma irradiation.

Animals were subjected to total-body irradiation using a Cesium-137 irradiator (Mark I irradiator, JL Shepherd & Associates) calibrated to a dose of 12 Gy. All procedures followed the biosafety guidelines of Rutgers New Jersey Medical School.

### Tamoxifen administration.

Forty mg/kg body weight of tamoxifen in corn oil was injected into mice of 8–12 weeks of age intraperitoneally as determined by the experimental scheme. Depending on the experiments, 4–7 days after tamoxifen administration, intestinal tissues were collected for analysis (118). For linage trancing after irradiation, a single dose of tamoxifen was administrated to mice 3 days after irradiation to trace green stripe formation by Lgr5 cells that were either Cdc42 deficient or V2-overexpressing. These mice were sacrificed 7 days after irradiation for the analysis of linage tracing and mitotic events.

### Administration of EGF to mice.

Recombinant murine EGF (315-09 B, PeproTech) was injected intraperitoneally at a concentration of 1 μg/g body weight, 30 minutes before tissue collection.

### EdU and BrdU incorporation in mice.

Mice were intraperitoneally injected with BrdU labeling reagent (00-0103, Invitrogen, Thermo Fisher Scientific) at 10 μL/g body weight 30 minutes before tissue collection. EdU was injected intraperitoneally into mice at 100 mg/kg 2, 6, or 24 hours before tissue collection. Intestinal tissue was embedded in either paraffin or OTC (Thermo Fisher Scientific) for sectioning. BrdU was detected by BrdU antibody and secondary fluorescent anti-rat antibody. EdU was detected by a Click-iT EdU Imaging Kit (C10338, Invitrogen, Thermo Fisher Scientific).

### Immunofluorescence and immunohistochemistry.

Intestinal tissues were collected and fixed in 4% paraformaldehyde or 10% neutral formalin buffer and embedded in paraffin. Sections (5 μm) were sliced, dewaxed, and subjected to antigen retrieval (0.1 M citric acid, pH 6.0, for most of the antibodies except p-Stat3 using DAKO Target Retrieval Solution and Signal Stain EDTA Unmasking Solution, respectively). Slides were immersed into antigen retrieval buffer at a sub-boiling temperature for 15 minutes. After incubation with 3% H_2_O_2_ in methanol for 10 minutes, sections were blocked in PBS containing 0.1% Triton X-100, 2% BSA, and 2% normal serum for at least 1 hour at room temperature, then probed with indicated antibodies at 4°C overnight. The next morning, slides were washed in PBS 3 times and probed with biotinylated secondary antibody (for IHC) or fluorescence-conjugated secondary antibodies (for immunofluorescence). After 1-hour incubation at room temperature, slides were washed 3 times. For IHC, slides were incubated with streptavidin-conjugated horseradish peroxidase (HRP) for an hour at room temperature. DAB HRP substrate kit was used for development. For immunofluorescence, slides were washed with PBS 3 times and subsequently subjected to DAPI counterstaining, before being air-dried and mounted with ProLong Gold antifade medium (Thermo Fisher Scientific). Immunofluorescence images were collected by LSM 510 Laser Scanning Microscope and analyzed by AIM software (version 4.2) or Fiji software (https://fiji.sc).

### Enteroid culture, 4-OHT treatment ex vivo, propidium iodide staining, and CellTiter-Glo 3D cell viability assay.

Enteroid cultures from Cdc42-deficient or Cdc42-WT mice were conducted based on previously described methods (119). To induce Cdc42 deletion in iKO enteroids, 100–200 WT or *Cdc42^iKO^* crypts were seeded in Matrigel with ENR medium and allowed to develop for 3 days. On day 4, 500 nM 4-OHT was added to the enteroids for 24 hours. Viable enteroids were counted under a bright-field microscope 1–7 days after 4-OHT treatment. For propidium iodide staining, enteroids were incubated with 500 nM propidium iodide (P4170, MilliporeSigma) in PBS solution at 37°C for 10 minutes and washed with PBS before imaging. For 3D cell viability assay, 100 organoids were seeded in each well on a 96-well plate and cultured in ENR medium for 4 days before addition of 4-OHT. Cell viability was determined typically at 72 hours after 4-OHT treatment using the Cell Titer-Glo 3D Cell Viability Assay (G9681, Promega) by Glomax system (E9032, Promega).

### Embedding enteroids for histological analysis.

To dissolve Matrigel-containing enteroids, medium was removed from wells and treated with 500 μL of Corning Recovery Solution (354253, Corning) on ice for 10 minutes. A p1000 pipette was then used to dissolve remaining Matrigel. Then, 2–3 wells of enteroids were combined in a microcentrifuge tube and centrifuged at 200 *g* for 5 minutes. Ice-cold PBS was used to wash the pellet. After a 2-minute centrifugation at 200 *g*, PBS was removed, and enteroids were resuspended in 4% paraformaldehyde for 10–15 minutes. After fixation, enteroids were centrifuged at 200 *g* for 5 minutes and washed 3 times in cold PBS. After removing the last PBS wash, 10–20 μL of Matrigel was added to form a clump of enteroids and left at 37°C to solidify. Once solid, 70% ethanol was added and stored at 4°C until paraffin embedding at the Histology Core of Rutgers New Jersey Medical School.

### CDC42 G-LISA assay.

Caco2 cells were serum starved overnight and then treated with EGF (50 ng/mL, 15-09 B, PeproTech), Wnt3a (100 ng/mL, 315-20, PeproTech), TGF-α (50 ng/mL, T7924, MilliporeSigma), Noggin (100 ng/mL, 250-38, PeproTech), and R-Spondin (1 μg/mL, 3474-RS-050, R&D Systems, Bio-Techne), respectively. CDC42 activity was determined before and after treatment by CDC42-specific G-LISA assay (BK127, Cytoskeleton) performed according to the manufacturer’s instructions.

### Lentivirus-mediated Cdc42 knockdown.

To stably knock down Cdc42 in Caco2 cells, human CDC42-specific lentiviral transduction particles (TRCN0000047628, MilliporeSigma) were added to cells at 1/5 multiplicity of infection with 8 μg/mL polybrene. Cells infected by non-Target shRNA Control Transduction Particles (SHC203V, MilliporeSigma) were used as control. Infected cells were selected by 10 μg/mL puromycin for 2 weeks. To induce MAPK activation, 100 ng/mL human EGF or 100 ng/mL mouse recombinant Wnt3a was added to serum-starved cells for the indicated time before cells were harvested for p-ERK detection.

### Transfection of HEK293 cells.

HEK293 cells were prepared to reach 70%–90% confluence at the time of transfection. Two micrograms plasmid DNA was transfected using Lipofectamine 3000 (L3000008, Invitrogen, Thermo Fisher Scientific). Forty-eight hours after transfection, cell lysate was made for further experiments.

### Atomic force microscope analysis.

A commercial atomic force microscope system (Dimension ICON, Bruker-Nano) was used to measure all the cellular surface plasma membrane properties. As previously published (42), an MLCT-C cantilever was used to measure the Young’s modulus of cells, with a spring constant at 0.01 N/m calibrated via the thermal tune method (Bruker-Nano). To avoid the substrate effect and keep consistency of the measurement conditions, all the measurements were performed at the top of the cell over the cell nucleus. The probe radius was calibrated by using a tip-radius calibration sample, and a silicon sample was used as the hard reference sample for all the indentation measurements. To minimize the cantilever drift due to the temperature fluctuation caused by the heating of the laser, the system was thermally equilibrated at 37°C for 40 minutes before the measurements.

### Proteomic analysis with mass spectrometry.

HEK293 cells were transfected with empty pQCXIP-3×Flag vector, pQCXIP-3×Flag-Cdc42-V1, or pQCXIP-3×Flag-Cdc42-V2. Cell lysates were subjected to immunoprecipitation using a Flag antibody (FLAGIPT1, MilliporeSigma). Immunoprecipitates were resolved by NuPAGE 4%–12% Bis-Tris Protein Gels (NP0335, Thermo Fisher Scientific), fixed for 1 hour with fixative containing 50% methanol and 10% acetic acid. After staining with Ruby Red, protein bands were cut for mass spectrometry analysis at Center for Advanced Proteomics Research of Rutgers University. Data were analyzed with Scaffold 4 software, Database for Annotation, Visualization and Integrated Discovery Bioinformatics Resources 6.8, Excel 3D Scatter Plot v2.1, and esyN (120).

### Phospho-kinase array analysis.

Lysates from HEK293 cells transfected with empty vector, Cdc42-V1, or Cdc42-V2 were subjected to a kinase array analysis using the Proteome Profiler Human Phospho-Kinase Array (ARY003B, R&D Systems, Bio-Techne) according to the manufacturer’s instructions.

### OptiPrep gradient cellular fractionation.

HEK293 cells were lysed with buffer composed of 150 mM NaCl, 50 mM Tris-HCl pH 8.0, 5 mM EDTA, and 0.5% Triton X-100, supplemented with protease and phosphatase inhibitors. After solubilization for 20 minutes at 4°C, 300 μL of lysates were combined with 600 μL of 60% OptiPrep solution to yield a 900 μL 40% OptiPrep-lysate mixture. After loading this mixture into the bottom of a Beckman ultracentrifuge tube, 3 mL of 30% OptiPrep solution (in lysis buffer) and 750 μL of 15% OptiPrep solution was overlaid sequentially, followed by 500 μL of pure lysis buffer on the top. Samples were centrifuged at 427,914 *g* for 3.5 hours in rotor SW41Ti at 4°C. After taking two 800 μL fractions from the top, seven 0.5 mL fractions were sequentially collected from the surface layer toward the bottom of the tube and used for Western blot. Lysates collected at the interface of 15% and 30% were referred to as the detergent-resistant fraction (or lipid raft). The rest of fractions were referred to as detergent soluble.

### Real-time PCR.

Total mRNAs were extracted using QIAGEN RNeasy Mini Kit from scraped epithelia. cDNA synthesis was performed using Thermo Fisher Scientific Maxima H Minus First Strand cDNA Synthesis Kit. RT-PCR reactions were assembled using the SYBR Green Real-Time PCR Master Mixes (Thermo Fisher Scientific). Reactions were run in replicates by a Roche LightCycler 480. The PCR cycling conditions were 10 minutes preheating at 95°C followed by 45 cycles of denaturing (95°C for 10 seconds), annealing (60°C for 10 seconds), and extension (72°C for 10 seconds). Fluorescent signal was acquired during the extension phase. Melting curves were analyzed to ensure PCR specificities. Housekeeping genes (such as HPRT) were used as internal references. For detection of V1- or V2-specific CDC42 mRNA levels in human colorectal cancers, specific TaqMan probes (ID: Hs03044122_g1 for V1; ID: Hs00741586_mH for V2) were used in TaqMan quantitative PCR on RNAs of paired human colorectal tumor and adjacent normal tissues (OriGene TissueScan, colon cancer cDNA array III; HCRT103). Primer sequences used in this study can be found in Supplemental Table 3.

### Bulk RNA-Seq and GSEA.

RNA-Seq data were deposited at National Center for Biotechnology Information’s BioProject with accession number GSE124848. GSEA (121, 122) used preranked files of differentially expressed genes calculated by: rank metric = –log(*P* value) × SIGN(logFC) (123). GSEA was performed on ISC gene signatures (124). Normalized enrichment scores and *P* values were documented (Kolmogorov-Smirnov test). Heatmaps of select EGFR and cytokine genes were generated by plotting *Z* scores of fragments per kilobase million normalized RNA-Seq data of individual replicates.

### Statistics.

Statistical and graphic data analysis was conducted using GraphPad Prism 7.04 (https://www.graphpad.com) and Microsoft Excel 2016. The data were presented as mean ± SEM in bar graphs or in box-and-whisker plots. The histology and immunostaining results were reported from 3 to 20 sections of at least 3 mice in each experiment unless stated differently. Each experiment had technical replicates that were reported in graphs to demonstrate intrasample variations. To quantify immunostaining results, ImageJ (NIH, 1.6.0_24 version) IHC toolbox package (https://imagej.nih.gov/ij/plugins/ihc-toolbox/index.html) was used to determine signals within a crypt or a crypt-villus unit depending on the specific antigen and experiment elaborated in figure legends. At least 20 crypt or villus units were analyzed from each section. Animal numbers used in each experiment were detailed in figure legends. Two-tailed paired Student’s *t* test was used for experiments containing 2 groups unless specifically stated otherwise. A *P* value less than 0.05 was considered significant in these *t* tests. For animal experiments with more than 2 groups, 1-way ANOVA and multiple comparisons were used, where Bonferroni’s correction was applied to justify the significance. As a result, in Figure 4, G, H, K, and L; and Figure 7, E and G, *P* < 0.025 was considered significant, and for Figure 5, D, F, G, I, and J; and Figure 6, B, C, E, G, and I, *P* < 0.0167 was considered significant.

### Study approval.

Experimental procedures in this study were approved by the Institutional Animal Care and Use Committee of Rutgers University. Because human cDNAs were commercially purchased, no institutional permission was necessary as they were deidentified samples.

## Author contributions

XZ, NG, and III conceived the idea. NG, GY, QZ, PK, RC, MPV, and III supervised the study. XZ, SB, LPA, KT, JF, DL, YZ, JW, RF, LZ, WH, and EMB performed experiments. XZ, EMB, and NG wrote the manuscript.

## Supplementary Material

Supplemental data

## Version 1 07/20/2020

In-Press Preview

## Version 2 08/20/2020

Electronic publication
