# Supplementary material for: Elevating EGFR-MAPK program by a nonconventional Cdc42 enhances intestinal epithelial survival and regeneration
Source: JCI Insight. 2020 Aug 20;5(16):e135923. doi: 10.1172/jci.insight.135923 (PMC7455142; doi:10.1172/jci.insight.135923)

SUPPLEMENTARY INFORMATION:

Elevating EGFR-MAPK program by a non-conventional Cdc42 enhances intestinal epithelial survival and regeneration

Zhang et al.

Suppl. Table 1. Cdc42-interacting proteins identified in Cdc42 V1 and V2 proteomics.

Proteins Identified	Spectra counts (V1)	Spectra counts (V2)	Unique Peptide (V1)	Unique Peptide (V2)
Desmoplakin	150	52	82	36
Actin, cytoplasmic 1	89	49	1	1
Actin, cytoplasmic 2	89	49	19	16
Ras GTPase-activating-like protein IQGAP1	84	68	50	49
Junction plakoglobin	65	20	29	14
Desmoglein-1	60	17	24	10
Ras GTPase-activating-like protein IQGAP2	45	35	35	28
Protein phosphatase 1B	44	25	16	11
Tubulin alpha-1B chain	42	31	17	15
Tubulin alpha-1A chain	38	29	21	18
Rho GDP-dissociation inhibitor 1	34	10	10	6
Myosin-9	32	13	27	12
Cell division control protein 42	30	12	10	4
14-3-3 protein epsilon	27	13	11	5
TGF-beta-activated kinase 1 and MAP3K7-binding protein 1	24	19	13	10
Mitogen-activated protein kinase kinase kinase 7	17	8	11	6
Annexin A1	17	12	10	11
Clathrin heavy chain 1	14	7	12	7
Serine/threonine-protein kinase 38	14	5	9	4
Myosin light polypeptide 6	10	3	6	2
Proliferating cell nuclear antigen	10	9	7	7
Myosin-10	8	2	6	2
Mitogen-activated protein kinase 1	7	3	5	3
Ras GTPase-activating-like protein IQGAP3	7	8	2	6
Dynactin subunit 2	4	3	2	1
Serine/threonine-protein kinase PAK 4	4	1	3	1
Growth factor receptor-bound protein 2	4	1	4	1
Annexin A11	4	2	4	2
AP-2 complex subunit alpha-1	3	1	3	1
Actin-related protein 2/3 complex subunit 2	3	1	3	1
Actin-related protein 2/3 complex subunit 4	3	1	3	0
AP-2 complex subunit beta	2	1	2	1

Supplementary Figure Legends:

Suppl. Fig. 1

(A) Bright field images of representative of WT and Cdc42$^{\Delta IEC}$ enteroids.

(B) Bright field images of representative WT and iKO enteroid after 72 hours of 4-OHT treatment.

(C) Enteroids from WT or Cdc42$^{\Delta IEC}$ mice were treated with or without CHIR99021 for 24 hours immediately after seeding to Matrigel. Quantification of enteroid viability was from 3 independent experiments.

(D) Cdc42 GLISA assay based on OD490 luminescence showed that Cdc42 activity was increased with different intensities in serum starved Caco2 cells by EGF, TGFa, Wnt3a, Noggin, R-spondin, or a combination of Wnt3a and EGF. Reading of OD490 were collected at 0 min (without growth factors), 1.5 min, 3 min, 6 min and 12 min after addition of growth factor.

(E) 3D representation of Flag-Cdc42 V1 and V2 interactomes identified in the proteomic analysis. Purple and red dots represent V1- and V2-specific interactor while gray dots are proteins associated with both. Note that a single dot might represent multiple proteins having identical numbers of peptide spectrum.

(F) List of Cdc42-assocated proteins, detected by proteomic analysis, which are known to mediate clathrin endocytosis, regulate actin organization, and affect MAPK signaling.

(G) Phospho-kinase array analysis of 43 kinase targets in Cdc42-V1 or V2 overexpressing HEK293T cells compared to empty vector transfected cells.

(H) Co-immunoprecipitation using HEK293T cell lysates overexpressing empty vector, Flag-V1 or Flag-V2 showed that PAK1 bound equally to both Cdc42 variants in soluble cellular fractions. R, detergent-resistant; S, soluble.

Suppl. Fig. 2

(A) Immunohistochemistry for cleaved caspase 3 on enteroids at indicated time points after 4-OHT treatment. Red arrows pointed to cleaved caspase 3+ cells.

(B) Quantification of percentage of cells showing cleaved caspase 3 staining at indicated time points after 4-OHT addition.

(C) EasN analysis of Cdc42 interactomes showed protein networks known to involve in EGFR endocytosis and MAPK signaling.

Suppl. Fig. 3

(A) RT-PCR detection of mRNAs of both Cdc42 variants in mouse enteroids.

(B) Cdc42 V2 specific antibody detected V2 expression in WT fetal mouse intestines. HEK239T cells overexpressing V2 was used as a positive control.

Suppl. Fig. 4.

(A-B) Quantification of intestinal villus length and crypt number from 3 animals per genotype.

(C) Immunohistochemistry of Cox1+ cells, with red arrowhead pointing at Cox1+ cells.

(D-E) Immunohistochemistry for DCLK1+ Tuft cells and quantification from 3 animals per genotype.

(F) High magnification images of Alkaline phosphatase (AP) staining showed representative AP+ inclusion bodies (arrows) in KO IECs.

Suppl. Fig. 5

(A) qRT-PCR for ISC markers in V2^{Tg} mice relative to WT mice showed significant increase of Bmi1, Hopx and Lgr5. N=3 for each genotype.

(B) Western blots showed increased β-catenin protein level in V2^{Tg} small intestine. Decreased β-catenin expressions in KO mouse ileum and colon were rescued by Cdc42-V2 in Cdc42KO; V2^{Tg} mice.

(C) Immunohistochemistry for β-catenin showed increased nuclear β-catenin in V2^{Tg} crypt cells. N=2 for each genotype.

Suppl. Fig. 6

(A-B) Representative images and quantifications showing the gross intestinal morphology of designated genotypes 7 days after irradiation. Note a clear elongation of V2^{Tg} gut length and a severe shortening of KO gut.

(C) H&E stained post-irradiation ileal sections of designated genotypes 3 days after irradiation.

(D) IHC images of Olfm4+ crypts 3 days post irradiation.

(E) Olfm4+ crypts were quantified from multiple ileal sections of 3 post-irradiation animals per genotype.

KEY RESOURCES TABLE

REAGENT or RESOURCE	SOURCE	IDENTIFIER
Antibodies		
Rabbit anti-p-ERK	Cell Signaling	9101s
Rabbit anti-ERK	Cell Signaling	4695s
Mouse anti-β-Catenin	BD Biosciences	610153
Rabbit anti-p-Lrp6	Cell Signaling	2568s
Rabbit anti-Lrp6	Cell Signaling	3395S
Mouse anti-β-actin	Santa Cruz	sc-4778
Rabbit anti-Clathrin	Cell Signaling	4796p
Mouse anti-α-Tubulin	Sigma-Aldrich	T6199
Rabbit anti-EGFR	Santa Cruz	sc-03
Rabbit anti-Cdc42	abcam	ab64533
Rabbit anti-p-EGFR^{Y1068}	Cell Signaling	2234s
Rabbit anti-PAK1	Cell Signaling	2602s
Rabbit anti-Fyn	Cell Signaling	4023
Mouse anti-Flotillin1	BD Biosciences	610820
Rabbit anti-Caveolin1	Cell Signaling	3267s
Rabbit anti-Calnexin	cell signaling	2433
Rabbit anti-PI3K-P110	Cell Signaling	4255
Rabbit anti-p-ERBB2$^{Y1221/1222}$	Cell Signaling	2243T
Rabbit anti-ERBB2	Cell Signaling	4290T
Goat Anti-DDDDK tag	abcam	ab95045
Mouse anti-pHH3	Cell Signaling	9706s
Rabbit anti-Cdc42V2	El-Husseini, A.	(Kang et al., 2008)
Rabbit anti-Cdc42V2	LifeSpan BioSciences	LS-C153281
Rabbit anti-EEA1	Cell Signaling	3288s
Rabbit anti-EGFR	Cell Signaling	4267p
Mouse anti-Flag	Sigma-Aldrich	f1804
Rabbit anti-Ki67	abcam	ab16637
Goat anti-Lysozyme C	Santa Cruz	sc-27958
Goat anti-GFP	abcam	ab6673
Rabbit anti-Cleaved Caspase3	Cell Signaling	9664s
Mouse anti-E-Cadherin (clone 36)	BD Biosciences	610182
Rabbit anti-Olfm4 (clone D6Y5A)	Cell Signaling	39141
Rabbit anti-lysozyme	Biogenex	AR024-10R
Rat anti-BrdU	Accurate Chemical	OBT0030G

Donkey anti-Rabbit, Alexa Fluor 488	Thermo Fisher Scientific	A-21206
Donkey anti-Rabbit, Alexa Fluor 546	Thermo Fisher Scientific	A10040
Donkey anti-Goat, Alexa Fluor 488	Thermo Fisher Scientific	A-11055
Donkey anti-Goat, Alexa Fluor 555	Thermo Fisher Scientific	A-21432
Donkey anti-Rat, Alexa Fluor 488	Thermo Fisher Scientific	A-21208
Donkey anti-Mouse, Alexa Fluor 555	Thermo Fisher Scientific	A-31570
Donkey anti-Mouse, Alexa Fluor 647	Thermo Fisher Scientific	A-31571
Biotinylated Horse Anti-Rabbit IgG	Vector Laboratories	BA-1100
Chemicals and Recombinant Proteins		
propidium iodide	Sigma-Aldrich	P4170
CHIR99021	Sigma-Aldrich	SML1046-5MG
Corn oil	Sigma-Aldrich	C8267
Tamoxifen	Sigma-Aldrich	T5648
Citric acid	Sigma-Aldrich	251275
Donkey serum	Sigma-Aldrich	D9663
Bovine Serum	Sigma-Aldrich	A3294
EdU (5-ethynyl-20-deoxyuridine)	Thermo Fisher Scientific	A10044
BrdU	Invitrogen	00-0103
Recombinant Murine Wnt-3a	Peprotech	315-20
Recombinant Murine EGF	Peprotech	315-09 B
Recombinant Murine Noggin	Peprotech	250-38
Recombinant Murine R-spondin	R&D	3474-RS-050
Critical Commercial Assays		
CDC42-specific GLISA assay	Cytoskeleton	BK127
Maxima Universal First Strand cDNA Synthesis Kit	Thermo Fisher Scientific	K1671
Cell Titer-Glo 3D Cell Viability Assay	Promega	G9681
Flag Immunoprecipitation Kit	Sigma-Aldrich	FLAGIPT1
GoTaq Probe qPCR Master Mix	Promega	A6101/2
Advanced DMEM/F12 medium	Thermo Fisher Scientific	12634028
Corning Recovery Solution	Corning	354253
4’,6-Diamidino-2-Phenylindole (DAPI)	Thermo Fisher Scientific	D1306
SignalStain EDTA Unmasking Solution	Cell Signaling	14747
DAKO Target Retrieval Solution	Agilent	S1699

Prolong Gold antifade medium	Thermo Fisher Scientific	P36930
VECTASTAIN Elite ABC HRP Kit (Peroxidase, Standard)	Vector Laboratories	PK-6100
RNeasy Plus Micro kit	QIAGEN	74034
DAB Peroxidase (HRP) Substrate Kit (with Nickel), 3,30 -diaminobenzidine	Vector Laboratories	SK-4100
Click-iT EdU Alexa Fluor 555 Imaging Kit	Thermo Fisher Scientific	C10338
Experimental Models, Cell lines		
Cdc42 flox	Brakebusch, C.	(Wu et al., 2006)
Cdc42 V2^{Tg}	This study	
Villin-CreER	Robine, S.	(El Marjou et al., 2004)
Rosa-ZsGreen	Jackson Lab	007906
emEGFR	Coffey, R.	(Yang et al., 2017)
B6.129P2-Lgr5$^{tm1(cre/ERT2)Cle}$/J	Jackson Lab	008875
Human HEK293	ATCC	CRL-1573
Human Caco2	ATCC	HTB-37
Software and Algorithms		
Prism	GraphPad	https://www.graphpad.com/scientific-software/prism/
Fiji software	(Schindelin et al., 2012)	https://fiji.sc
Recombinant DNAs		
pcDNA6A-EGFR WT	Addgene	#42665
pcDNA6A-EGFR ICD (645-1186)	Addgene	#42667
pcDNA6A-EGFR ECD (1-644)	Addgene	#42666
CAG-3×Flag-Cdc42 V2	This study	
PQCXIP-3×Flag-Cdc42V2-K185R	This study	
PQCXIP-3×Flag-Cdc42V1	This study	
PQCXIP-3×Flag-Cdc42V2	This study	
CAG-lox-CAT-lox-unique Bam cloning site-hGH polyA (Clone 17)	Addgene	#53959
PQCXIP-3×Flag	This study	

SUPPLEMENTARY TABLE 1

PCR and RT-PCR primers used in this study.

Primer	Sequence
PCR--mouse cdc42 V1-F	TATGTATAGTCAGCGCGTGCCCCTGT
PCR--mouse cdc42 V1-R	CCACAGGCTGTCCTATGGTT
PCR--mouse cdc42 V2-F	GCGGAGAAGCTGAGGACAAGAT
PCR--mouse cdc42 V2-R	AAGAAGACGCAGAGGCTTTCA
Realtime PCR--mouse cdc42 V1/2-F	CACTCCAGAGACTGCTGAAAAGC

Realtime PCR--mouse cdc42 V1-R	TCATAGCAGCACACACCTGCG
Realtime PCR--mouse cdc42 V2-R	TGGGTTGAGTTTCCGGAGGC
Realtime PCR--mouse LRIG1-F	AAGGGAACTCAACTTGGCGAG
Realtime PCR--mouse LRIG1-R	ACGTGAGGCCTTCAATCAGC
Realtime PCR--mouse LGR5-F	ACCCGCCAGTCTCCTACATC
Realtime PCR--mouse LGR5-R	GCATCTAGGCGCAGGGATTG
Realtime PCR--mouse Bmi1-F	GGAGAAGAAATGGCCCACTACC
Realtime PCR--mouse Bmi1-R	TTGGCCTTGTCACTCCCAGA
Realtime PCR--mouse HopX-F	CATCCTTAGTCAGACGCGCA
Realtime PCR--mouse HopX-R	AGGCAAGCCTTCTGACCGC
Realtime PCR--mouse Olfm4-F	TGGCCCTTGGAAGCTGTAGT
Realtime PCR--mouse Olfm4-R	ACCTCCTTGGCCATAGCGAA
Realtime PCR--mouse Hprt-F	TCCCTGGTTAAGCAGTACAGC
Realtime PCR--mouse Hprt-R	TCCAACAAAGTCTGGCCTGT

References cited for experimental procedure

El Marjou, F., Janssen, K.-P., Hung-Junn Chang, B., Li, M., Hindie, V., Chan, L., Louvard, D., Chambon, P., Metzger, D., and Robine, S. (2004). Tissue-specific and inducible Cre-mediated recombination in the gut epithelium. genesis *39*, 186-193.

Kang, R., Wan, J., Arstikaitis, P., Takahashi, H., Huang, K., Bailey, A.O., Thompson, J.X., Roth, A.F., Drisdel, R.C., Mastro, R*., et al.* (2008). Neural palmitoyl-proteomics reveals dynamic synaptic palmitoylation. Nature *456*, 904-909.

Schindelin, J., Arganda-Carreras, I., Frise, E., Kaynig, V., Longair, M., Pietzsch, T., Preibisch, S., Rueden, C., Saalfeld, S., Schmid, B*., et al.* (2012). Fiji: an open-source platform for biological-image analysis. Nature methods *9*, 676-682.

Wu, X., Quondamatteo, F., Lefever, T., Czuchra, A., Meyer, H., Chrostek, A., Paus, R., Langbein, L., and Brakebusch, C. (2006). Cdc42 controls progenitor cell differentiation and β-catenin turnover in skin. Genes & Development *20*, 571-585.

Yang, Y.P., Ma, H., Starchenko, A., Huh, W.J., Li, W., Hickman, F.E., Zhang, Q., Franklin, J.L., Mortlock, D.P., Fuhrmann, S*., et al.* (2017). A Chimeric Egfr Protein Reporter Mouse Reveals Egfr Localization and Trafficking In Vivo. Cell Rep *19*, 1257-1267.

Zhang et al,. Supplement Figure 1

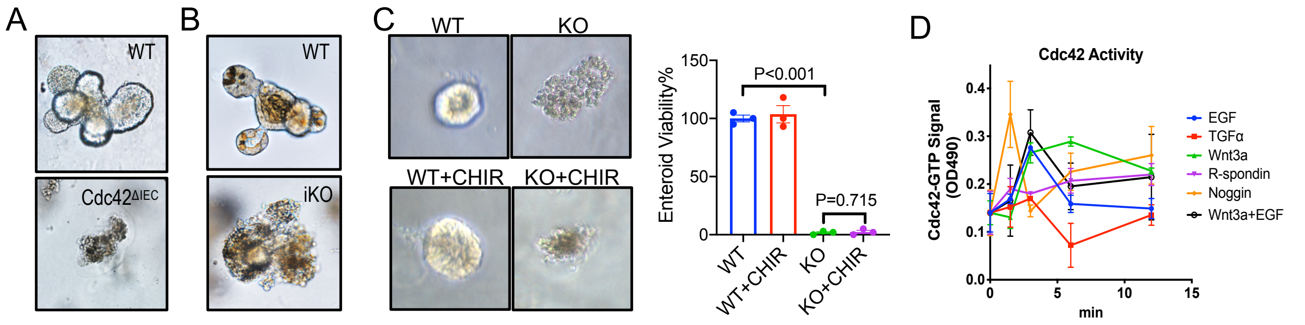

E

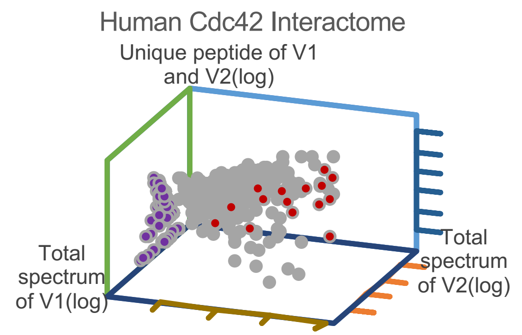

F

Function Clusters	Identified Proteins (275)	Spectra counts (Cdc42V1)	Spectra counts (Cdc42V2)
Cdc42	Cell division control protein 42 homolog	30	12
Formation of Clathrin-dependent endosomes	AP-2 complex subunit alpha-1	3	1
	AP-2 complex subunit beta	2	1
	AP-2 complex subunit sigma	1	0
	Clathrin heavy chain 1	14	7
	ADP-ribosylation factor 6	1	1
Regulation of Actin filament	Myosin light polypeptide 6	10	3
	Myosin regulatory light chain 12A	4	1
	Myosin-10	8	2
	Myosin-9	32	13
	Actin-related protein 2/3 complex subunit 2	3	1
	Actin-related protein 2/3 complex subunit 3	1	1
	Actin-related protein 2/3 complex subunit 4	3	1
	Actin, cytoplasmic 1	89	49
	Actin, cytoplasmic 2	89	49
	Actin-related protein 3	1	1
Components of MAPK endocytic signalosome	Ras-related protein Rab-5C	5	1
	Dynactin subunit 1	2	2
	Dynactin subunit 2	4	3
	Serine/threonine-protein kinase PAK 4	4	1
	Growth factor receptor-bound protein 2	4	1
	Mitogen-activated protein kinase 1	7	3
	Mitogen-activated protein kinase kinase kinase 7	17	8

G

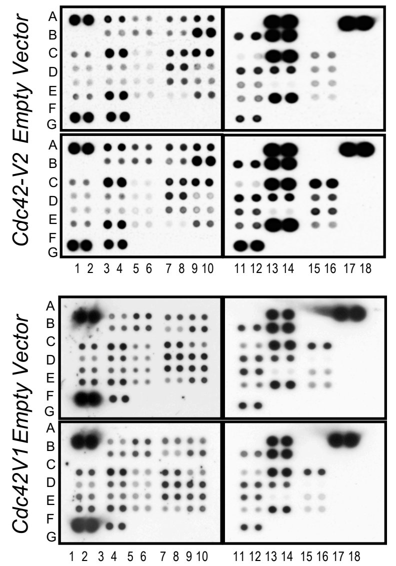

H

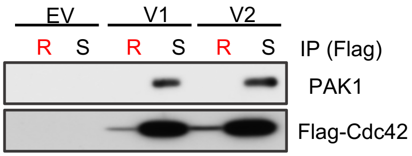

Zhang et al,. Supplement Figure 2

A

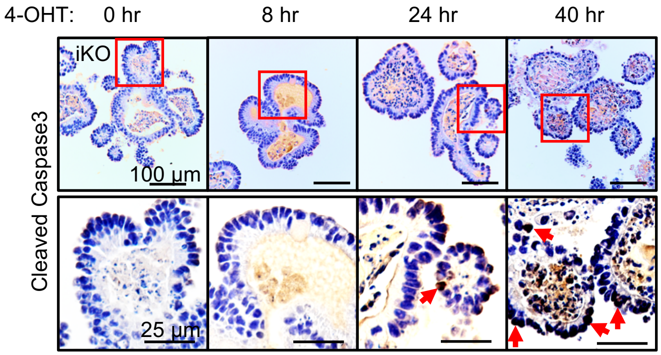

B

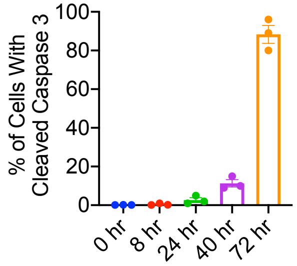

C

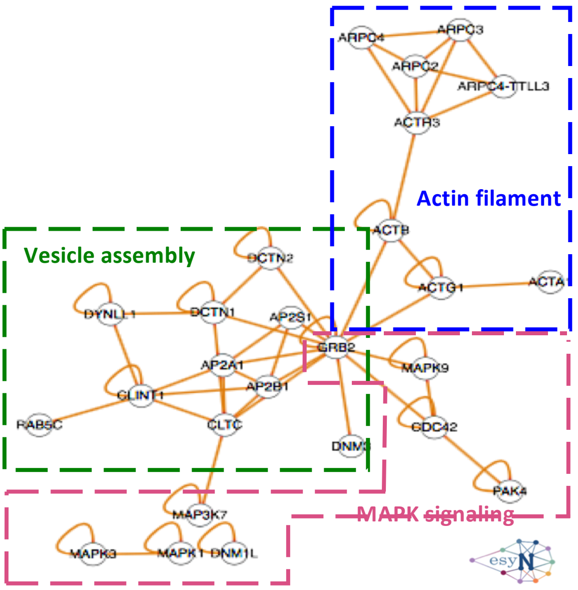

Zhang et al,. Supplement Fig. 3

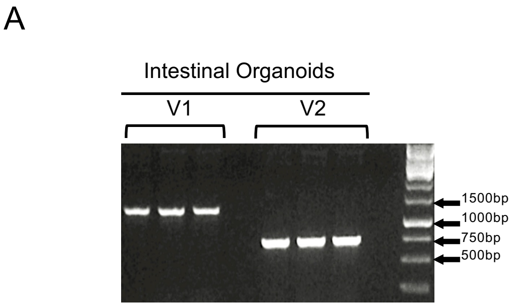

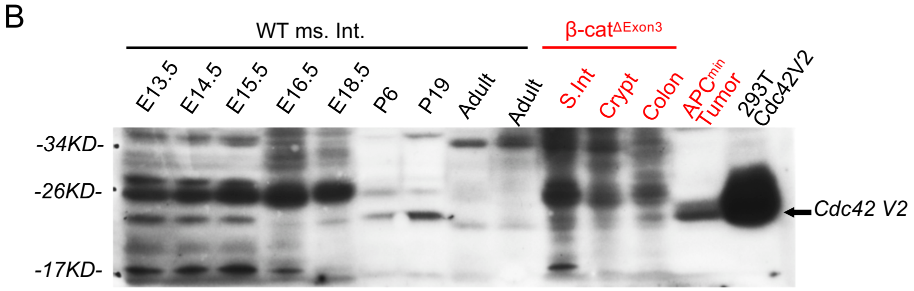

Zhang et al,. Supplement Fig. 4

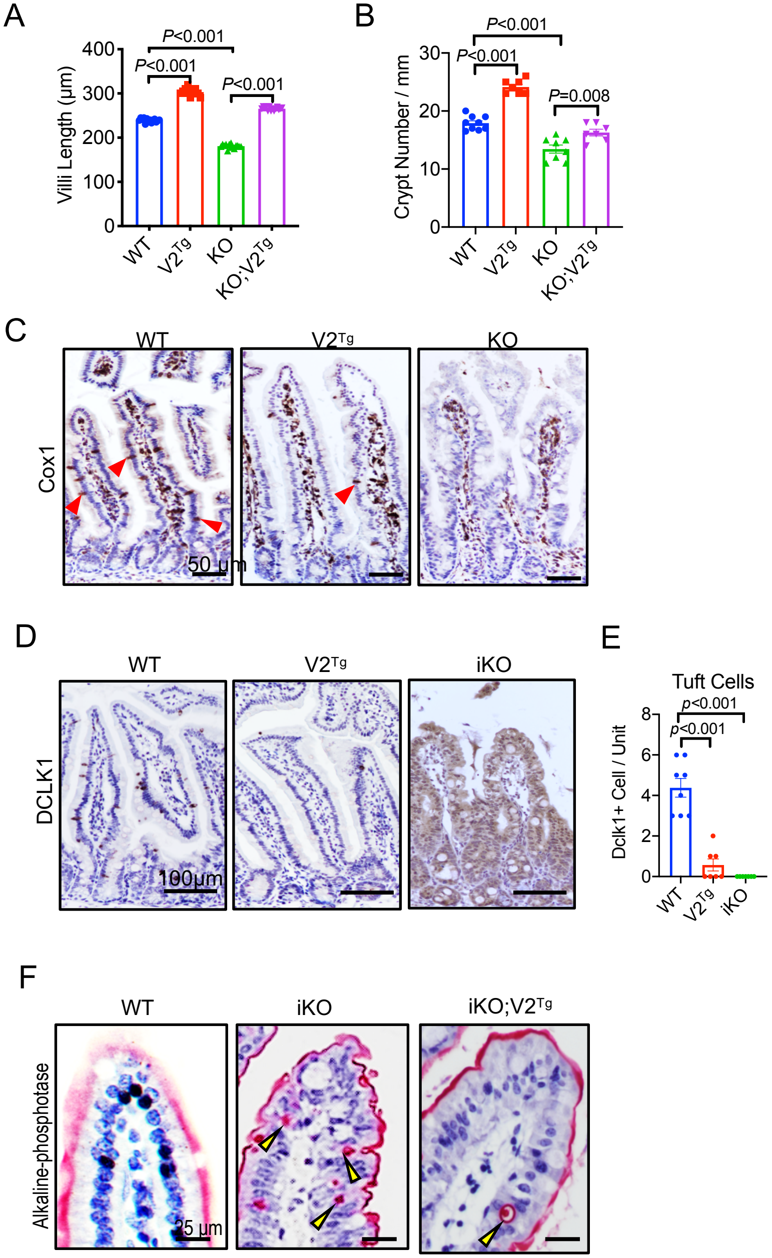

Zhang et al,. Supplement Fig. 5

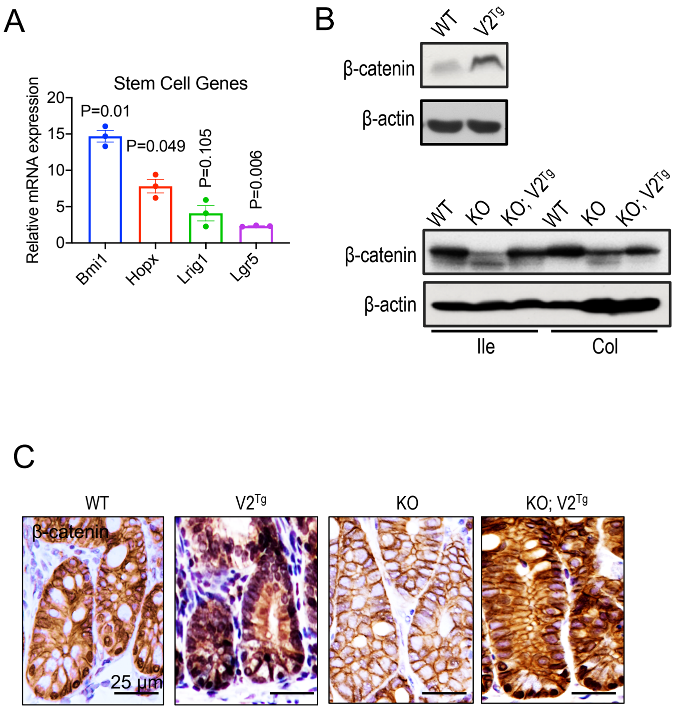

Zhang et al,. Supplement Fig. 6

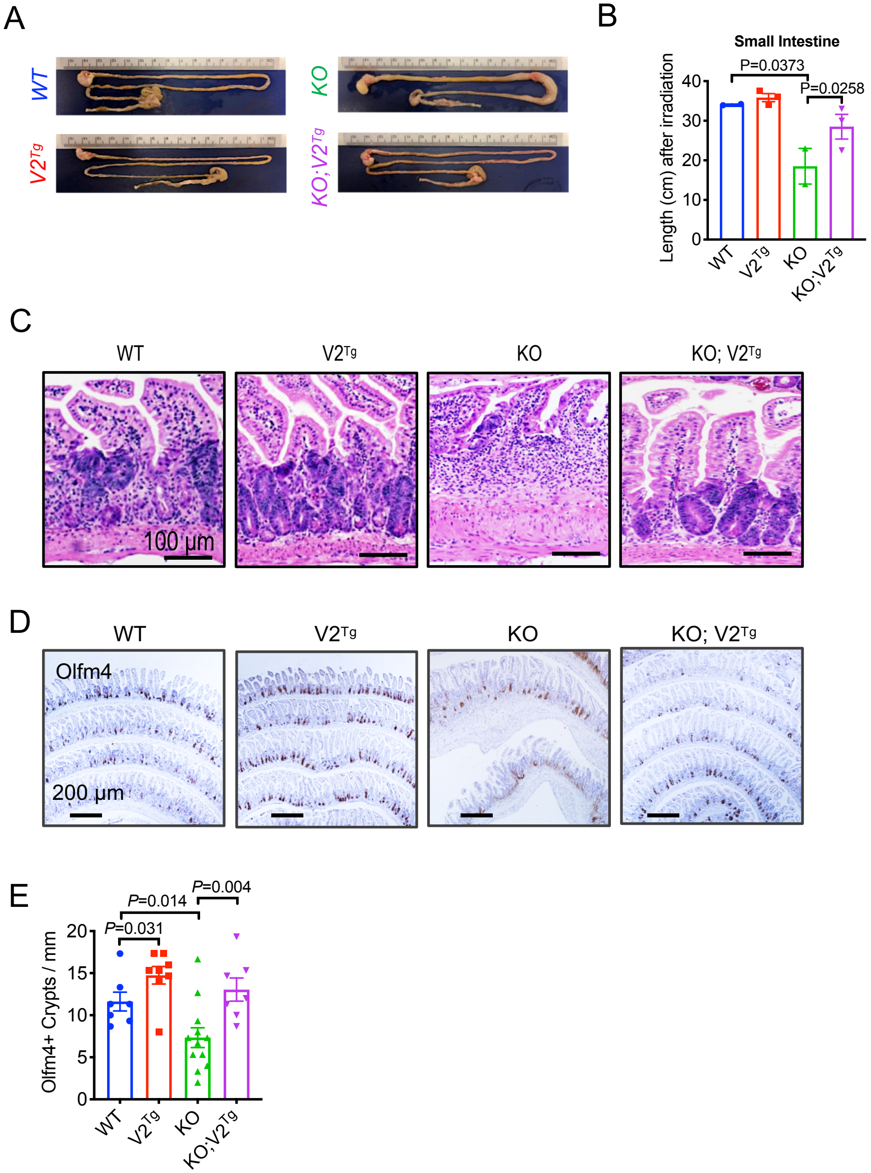

Supplement: Supplemental data [file jciinsight-5-135923-s045.pdf]
